# Restoring the epigenetically silenced PCK2 suppresses renal cell carcinoma progression and increases sensitivity to sunitinib by promoting endoplasmic reticulum stress

**DOI:** 10.7150/thno.48469

**Published:** 2020-09-15

**Authors:** Zhiyong Xiong, Changfei Yuan, Jian shi, Wei Xiong, Yu Huang, Wen Xiao, Hongmei Yang, Ke Chen, Xiaoping Zhang

**Affiliations:** 1 Department of Urology, Union Hospital, Tongji Medical College, Huazhong University of Science and Technology, Wuhan 430022, China.; 2 Institute of Urology, Union Hospital, Tongji Medical College, Huazhong University of Science and Technology, Wuhan 430022, China.; 3 Department of Pathogenic Biology, School of Basic Medicine, Huazhong University of Science and Technology, Wuhan 430030, China.; 4 Department of Nephrology, Union Hospital, Tongji Medical College, Huazhong University of Science and Technology, Wuhan 430022, P. R. China.

**Keywords:** PCK2, renal cell carcinoma (RCC), methylation, endoplasmic reticulum stress, Sunitinib sensitivity

## Abstract

**Rationale:** Tumors have significant abnormalities in various biological properties. In renal cell carcinoma (RCC), metabolic abnormalities are characteristic biological dysfunction that cannot be ignored. Despite this, many aspects of this dysfunction have not been fully explained. The purpose of this study was to reveal a new mechanism of metabolic and energy-related biological abnormalities in RCC.

**Methods:** Molecular screening and bioinformatics analysis were performed in RCC based on data from The Cancer Genome Atlas (TCGA) database. Regulated pathways were investigated by qRT-PCR, immunoblot analysis and immunohistochemistry. A series of functional analyses was performed in cell lines and xenograft models.

**Results:** By screening the biological abnormality core dataset-mitochondria-related dataset and the metabolic abnormality core dataset-energy metabolism-related dataset in public RCC databases, PCK2 was found to be differentially expressed in RCC compared with normal tissue. Further analysis by the TCGA database showed that PCK2 was significantly downregulated in RCC and predicted a poor prognosis. Through additional studies, it was found that a low expression of PCK2 in RCC was caused by methylation of its promoter region. Restoration of PCK2 expression in RCC cells repressed tumor progression and increased their sensitivity to sunitinib. Finally, mechanistic investigations indicated that PCK2 mediated the above processes by promoting endoplasmic reticulum stress.

**Conclusions:** Collectively, our results identify a specific mechanism by which PCK2 suppresses the progression of renal cell carcinoma (RCC) and increases sensitivity to sunitinib by promoting endoplasmic reticulum stress. This finding provides a new biomarker for RCC as well as novel targets and strategies for the treatment of RCC.

## Introduction

Cancer is a disease that is always accompanied by a variety of characteristic biological dysfunctions 1, 2. Renal cell carcinoma (RCC) is the most common lethal neoplasm of the urinary system, of which clear cell renal cell carcinoma (ccRCC) is the most prevalent pathological type, accounts for approximately 70-80% of all RCC cases 3, 4. RCC, in addition to the general biological abnormalities that are common to most tumors, is also known for its metabolic abnormalities 5. Although these biological dysfunctions are significant, many problems related to these dysfunctions have not been fully explained.

PCK2 (phosphoenolpyruvate carboxykinase 2) is a mitochondrial isoform of the key gluconeogenesis enzyme phosphoenolpyruvate carboxykinase (PEPCK) 6. It is widely involved in cell metabolism, including in physiological processes such as glucose metabolism and the tricarboxylic acid cycle (TCA), and plays an important role in maintaining cell homeostasis and activity 7, 8. Although some studies on PCK2 have been performed in the field of oncology, they have primarily focused on lung cancer and some gastrointestinal tumors 7, 9, 10, while none has focused on renal cell carcinoma.

The endoplasmic reticulum (ER) is widely involved in a variety of biological functions, including maintaining calcium homeostasis, mediating protein synthesis and transport, and regulating lipid and glucose metabolism. It constitutes a huge network connected to a variety of organelles in terms of function and physical structure 11. Thus, the endoplasmic reticulum (ER) is well positioned to integrate intracellular information, maintain intracellular homeostasis, and act as the center of cellular activity 12. When cell homeostasis is disrupted and the demand for ER function outweighs its capacity, endoplasmic reticulum stress (ER stress) is activated to restore balance 13. ER stress is a double-edged sword. When disruptions in the internal environment are relatively mild, ER stress can reverse those disruptions. However, when the body cannot correct these abnormalities, strong and chronic ER stress will induce apoptosis in the relevant cells 13. Similarly, ER stress also plays an important role in tumors, where it is highly correlated with tumor progression and treatment 14. Specifically, in RCC, ER stress can significantly inhibit tumor progression. A characteristic change of RCC is the large accumulation of lipids in cells. These lipids have been proved to play a vital role in the occurrence and progression of RCC and the key to their role is to maintain the stability of the endoplasmic reticulum by PLIN2. Once this stability is broken, thereby activating the endoplasmic reticulum stress, the progression of RCC will be significantly inhibited and lipid consumption will be accelerated 15. In addition, studies have shown that Cuprous oxide nanoparticles (CONPs) can inhibit the function of copper chaperone proteins, disturb copper trafficking, and activate the ER stress-induced apoptosis of renal cell carcinoma, thereby inhibiting the progression and alleviating drug resistance in renal cell carcinoma 16.

With the development of RCC treatments, anti-angiogenesis therapy has become the preferred drug treatment for RCC and of these treatments, sunitinib is the most commonly used clinical first-line drug 3. Although these drugs greatly improve the prognosis of patients with RCC, unfortunately, due to the presence of initial and acquired drug resistance, the disease always progresses after a median of 6-15 months, which results in a poor prognosis 17, 18. Clinically, once this drug resistance occurs, it will seriously endanger the lives of RCC patients. Despite the danger of this resistance, this phenomenon has not yet been resolved. Therefore, sunitinib resistance is still a challenge in RCC treatment and is a focus of RCC research.

In this study, PCK2 was found to be a novel biomarker that can predict the prognosis of RCC. It was also found that low expression of PCK2 in RCC was caused by methylation of its promoter region. At the same time, PCK2 was shown to inhibit RCC progression and alleviate sunitinib resistance. Mechanistic studies showed that this process is mediated by PCK2-activated ER stress. These findings provide a new biomarker for RCC as well as novel targets and strategies for the treatment of RCC.

## Methods

### Cell culture and reagents

The normal cell line HK2 and the human kidney cancer cell lines 786-0, A498, ACHN, CAKI, and OSRC were obtained from The American Type Culture Collection (ATCC, USA). Sunitinib-resistant kidney cancer cell lines were provided by the Department of Urology, Wuhan Union Hospital. Cells were cultured in an incubator at 37 ºC and 5% CO2 in DMEM high-glucose medium supplemented with 10% FBS and 1% penicillin-streptomycin.

The expression lentivirus for PCK2 and the corresponding control vector were purchased from Genechem, China. 5-AZA was obtained from MCE. ER-Tracker Red was obtained from Beyotime Biotechnology, China. Tauroursodeoxycholic Acid (TUDCA) was purchased from Selleckchem, S3654. The CRISPR/dCas9-mediated editing system for PCK2 specific demethylation was purchased from Genechem.

### Tissue samples

Thirty-two pairs of human RCC tissues were obtained from the Department of Urology, Union Hospital, Tongji Medical College (Wuhan, China) from 2017-2018. Adjacent normal tissues, which were located at least 5 cm away from the tumor site, were obtained and used as normal control tissues corresponding to the RCC tissues. And liquid nitrogen was used to store the tissues at -80 °C for RNA and protein extraction. All patients whose tissues were used were fully informed as to the content of the experiments. The Institutional Review Board of Huazhong University of Science and Technology approved the implementation of this study.

### Immunohistochemistry and immunofluorescence staining

For immunohistochemical staining, tissue samples embedded in formalin-fixed paraffin were first made into 4-μm sections. Subsequently, paraffin was removed using EDTA, rehydrated, and sections were incubated at 120 °C for 5 minutes for antigen retrieval. After completion, incubate for 15 minutes at room temperature with 3% H2O2. Sections were then blocked with fetal bovine serum. Next, the tissue sections were incubated overnight at 4 °C in primary antibody solution after which they were incubated with an appropriate HRP-conjugated secondary antibody for 1 h at room temperature. Finally, the immune complexes were visualized by DAB and sections were counterstained in hematoxylin.

To visualize the endoplasmic reticulum, ER-Tracker Red was used according to the manufacturer's instructions. Staining was captured using confocal microscopy, after which the acquired images were processed and quantified using ImageJ software.

### RNA isolation and real-time PCR analysis

According to the manufacturer's instructions, TRizol reagent (Thermo, Massachusetts, USA) was used to extract total RNA of tissues and cells. The purity and concentration of the RNA solution was measured using a NanoDrop 2000 spectrophotometer (NanoDrop Technologies, Wilmington, USA). In all, 1 μg of RNA was needed for reverse transcription. qPCR analysis was performed with a SYBR Green master mix (YEASEN, China) in StepOnePlus™ Real-Time PCR System (Thermo Fisher Scientific, USA). GAPDH was used to normalize the samples. The gene primers used are listed in table A.

### Methylation-Specific Polymerase Chain Reaction (PCR) (MSP)

Genomic DNA from RCC tissues was prepared following the manufacturer's instructions. The following MSP primers were designed according to the genomic sequences flanking the transcription start sites: PCK2-M-sense, 5'- GTTTGGAGTTTCGGGGTCGA -3'; PCK2-M-antisense, 5'- CGAAACGAACCACAACAAAAAC -3'; PCK2-U-sense, 5'- GGAGTTTGGAGTTTTGGGGTTG -3' and PCK2-U-antisense, 5'- AACAAAACAAACCACAACAAAAACT -3'. The primer sequences were oligosynthesized (Servicebio, China) for MSP to detect bisulfate-induced changes affecting unmethylated (U) and methylated (M) alleles.

### Bisulfite sequencing PCR (BSP)

Genomic DNA from RCC tissues and cell lines were prepared following the manufacturer's instructions. The following BSP primers were designed according to genomic sequences flanking the transcription start sites: PCK2-BSP-F, 5'-TAGTATTTTGGGAGGTTAAGGTAGGC -3'; PCK2-BSP-R, 5'-CTCCTCCTAATCAAAACTCCTTTCTAC -3'. Subsequently, carry out Bisulfite treatment of DNA, PCR amplification, Electrophoresis, Purify DNA from agarose gel, Transformation experiment using DH5α, PCR amplification identification, and DNA sequencing. Specific technical support is provided by Servicebio, China.

### Western Blotting assay

For the Western blot analysis, protein extraction was performed using RIPA protein lysis buffer (Beyotime Institute of Biotechnology, Haimen, China) containing a protease inhibitor cocktail and PMSF. The extracted protein was subjected to SDS-PAGE and transferred to a polyvinylidene fluoride (PVDF) membrane. The membrane was then blocked with 5% skim milk for 1 hour at room temperature. Subsequently, the primary antibody was used for incubation overnight. Prior to detection, membranes were washed with PBST and incubated with secondary antibodies in blocking buffer for 2 hours. The antibodies used for western blots were as follows: PCK2 ( Abclonal, A8446), IREa (CST, 3294S), P-IREa (Novus, NB100-2323), PERK (CST, 3192S), P-PERK (Abcam, ab192591), ATF6 (CST, 65880S), and GAPDH ( Proteintech, 60004-1-Ig).

### Cell viability assay

According to the manufacturer's instructions, 2 × 10^3^ cells were first seeded in a 96-well plate, and then a CCK8 assay (YEASEN, China) was used to test the cell proliferation capacity. Cell viability was measured at 0, 24, 48, 72, and 96 hours.

### Colony formation assay

In order to perform colony formation assays, 1,000 cells were first seeded into a 6-well plate, and cell colony formation was evaluated after 14 days of culture. The cells were then fixed in methanol during the assay, and then viable colonies (> 50 cells/colonies) were observed by staining with 0.05% crystal violet.

### Wound healing assay

When the cells reached 90% confluence in a 6-well plate in serum-free medium, a 10-µl pipette tip was used to generate a wound and was then washed with PBS to remove cell debris. Images were obtained at 0 and 24 hours after the injury. The images were processed by Photoshop 7.0 software.

### Transwell assay

A Transwell assay was used to assess the ability of cells to migrate and invade. The cells to be tested were first cultured in serum-free medium for 24 hours, after which they were seeded in a Transwell chamber. An additional 1: 8 ratio of Matrigel was added to evaluate the invasive capacity of the cells. After 24 hours, the cells were fixed in methanol and stained with 0.05% crystal violet. The resulting images were captured by a microscope, which randomly selected multiple fields for cell counting.

### *In vivo* tumor implantation

The 6-week-old male nude mice used in this study were purchased from Vital River Laboratory Animal Technology Co. Ltd. In all, 2 × 10^6^ cells were injected subcutaneously into the axilla of these mice to construct a xenograft tumor model. A metastasis model was constructed by injecting tumor cells into the tail vein to assess their metastatic ability. Tumor size was measured every 4 days. Immunohistochemical staining was performed according to a standard procedure as previously described. All animal experiments were performed in accordance with animal protocols approved by the Institutional Animal Use and Care Committee of Tongji Medical College, Huazhong University of Science and Technology.

### Bioinformatics Analysis

Data for genetic screening were obtained from the Oncomine database (https://www.oncomine.org) and the European Bioinformatics Institute (EMBL-EBI) (https://www.ebi.ac.uk). (Gene set (1) is derived from the mitochondrion (goterm) project in the "Gene array analysis of clear cell renal cell carcinoma tissue versus matched normal kidney tissue" data subset of the EMBL-EBI database; Gene set (2) is derived from the mitochondrion (goterm) project in the “Transcription profiling of clear cell renal carcinomas and normal kidney cortical tissues” data subset of the EMBL-EBI database; Gene set (3) is derived from the mitochondrion Go Cellular Component (GO) project in the “differentially expressed genes in renal cell carcinoma in Lenburg Renal” data subset of the Ocomine database; Gene set (4) is derived from the mitochondrion Go Cellular Component (GO) project in the “differentially expressed genes in renal cell carcinoma in Beroukhim Renal” data subset of the Ocomine database; Gene set (5) is derived from the Glucose metabolism (pathwayname) project in the “Gene array analysis of clear cell renal cell carcinoma tissue versus matched normal kidney tissue” data subset of the EMBL-EBI database; Gene set (6) is derived from the Glucose metabolism (pathwayname) project in the “Transcription profiling of clear cell renal carcinomas and normal kidney cortical tissues” data subset of the EMBL-EBI database). A clinical prognosis analysis of related molecules derived from data in the TCGA database (http://www.cbioportal.org/public-porta). CpG island predictive analysis was performed by http://www.urogene.org/. Gene function enrichment analysis was performed by gene set enrichment analysis (GSEA). STRING (https://string-db.org/) was used to create the interaction network of genes.

### Statistical analysis

All statistical analyses were performed using Excel 2016 (Microsoft) and SPSS Statistics 22.0 (IBM SPSS, Chicago, IL). All *in vitro* experiments were performed in triplicate and all data were represented as the mean ± SEM. Statistical analyses were performed using the Student *t* test and Pearson correlation coefficient. Univariate and multivariate Cox proportional hazard regression analyses were used to clarify the independent factors of RCC. The significance value was determined when *P* < 0.05.

## Results

### PCK2 was downregulated and predicted a poor prognosis in RCC

**Figure 1:**
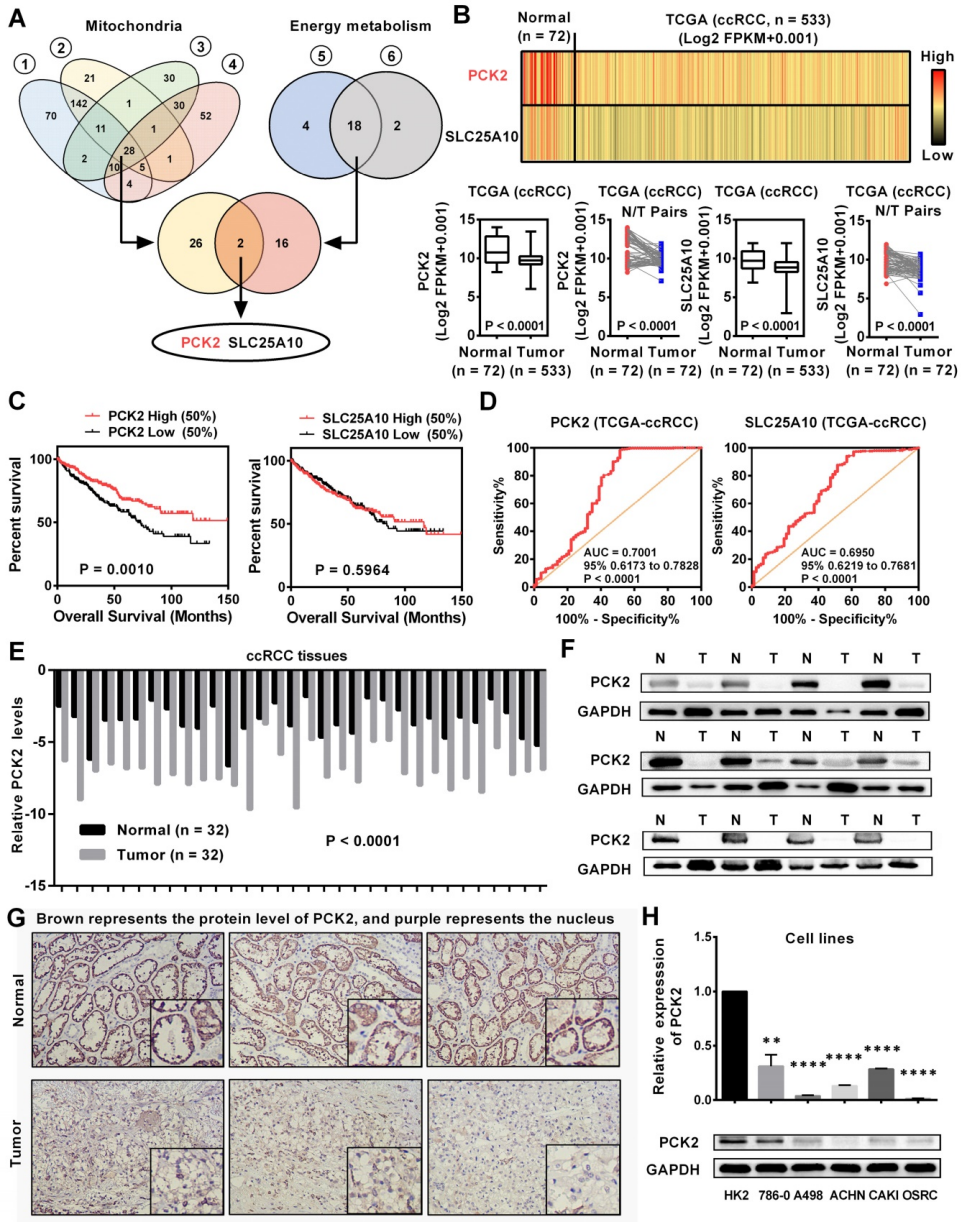
** PCK2 was downregulated and predicted a poor prognosis in RCC.** (A) Venn plots for mitochondrial metabolism related gene sets and energy metabolism related gene sets for screening differentially expressed genes. (Gene set (1 & 2) is derived from the mitochondrion (goterm) from EMBL-EBI; Gene set (3 & 4) is derived from the mitochondrion Go Cellular Component (GO) from Oncomine database; Gene set (5 & 6) is derived from the Glucose metabolism (pathway name) from EMBL-EBI) (B) mRNA levels of PCK2 and SLC25A10 in RCC tissues and paired normal tissues according to the data from the TCGA database. *t*-test, *P* < 0.0001. (C) Kaplan-Meier curves for overall survival (OS) as determined by PCK2 and SLC25A10 expression in RCC based on data from the TCGA database. (D) The ROC curves for PCK2 (AUC=0.7001 95% CI: 0.6173 to 0.7828; *P* < 0.0001) and SLC25A10 (AUC=0.6950 95% CI: 0.6219 to 0.7681; *P* < 0.0001) in RCC. (E) The mRNA levels of PCK2 in 32 RCC tissues and adjacent nonmalignant tissues. *t*-test, *P* < 0.0001. (F) The protein levels of PCK2 in RCC tissues and adjacent nonmalignant tissues. (G) Immunohistochemical (IHC) staining for PCK2 in RCC tissues and adjacent nonmalignant tissues (Brown represents the protein level of PCK2, and purple represents the nucleus). (H) Levels of PCK2 mRNA and protein in 5 RCC cell lines and a normal cell line. *t*-test, **P < 0.01, and ****P < 0.0001.

Tumors are accompanied by dysfunctions in a variety of biological processes. Previous studies have confirmed that mitochondria are the central organelles that mediate the functions of cell biology, and the disorders of mitochondrial function lead to abnormalities in various cellular biological functions 19. In addition, RCC is known for its significant metabolic abnormalities, in which energy metabolism is the center of these abnormalities 5. Although these abnormalities are significant, there are still many doubts about their relationship with tumors. To further elucidate the causes of these abnormalities in the biological functions in RCC, we screened a variety of public databases related to RCC. By screening the biological abnormality core dataset-mitochondria-related dataset and the metabolic abnormality core dataset-energy metabolism-related dataset in public database, two genes were found to be dysregulated in RCC (**Figure 1A, Table S1-5**). To further assess the expression of these two genes in RCC, we analyzed data from the TCGA-KIRC database which contains 533 ccRCC cases and 72 paired normal cases. As shown in **Figure 1B**, these two genes were both significantly downregulated in RCC and PCK2 has a relatively more pronounced low expression trend. Further bioinformatics analysis showed that PCK2 expression had a significant predictive value for the survival time of RCC patients, as lower PCK2 expression predicted a shorter overall survival time. However, no significant difference was observed in the survival times between those with high expression of SLC25A10 and those with low expression of SLC25A10 (**Figure 1C**). Subsequently, a ROC curve was used to assess the ability of these two genes to identify clinical RCC patients. The results showed that both genes could identify clinical RCC cases, and PCK2 has a higher AUC (area under the curve), which indicated that PCK2 had a relatively better diagnostic value for RCC (**Figure 1D**). Based on the above results, PCK2 was selected as a target molecule for further study. To further validate the value of PCK2 in RCC, we performed a more in-depth bioinformatics analysis. The low expression of PCK2 in RCC was confirmed again in four independent data sets from the Oncomine database (**Figure S1A**). The detailed analysis of the TCGA database also found that PCK2 not only predicted the survival time of RCC patients overall, but that it could also predict the survival time of patients in different clinical subgroups (**Figure S1B**). In addition, univariate and multivariate Cox regression analyses based on the TCGA database also confirmed that PCK2 is an independent prognostic marker for RCC (**Table 1**).

To verify the results of the bioinformatics analysis, we examined the expression of PCK2 at both the mRNA and protein levels in RCC tissues and cell lines. As shown in **Figure 1E-G,** the protein and mRNA levels of PCK2 were significantly lower in RCC tissues compared with normal tissues. Similar results were obtained in cell lines, in which the protein and mRNA levels of PCK2 were also greatly downregulated in the RCC cell lines compared with the control cell line (**Figure 1H**).

### Low expression of PCK2 in RCC was caused by DNA methylation

**Figure 2:**
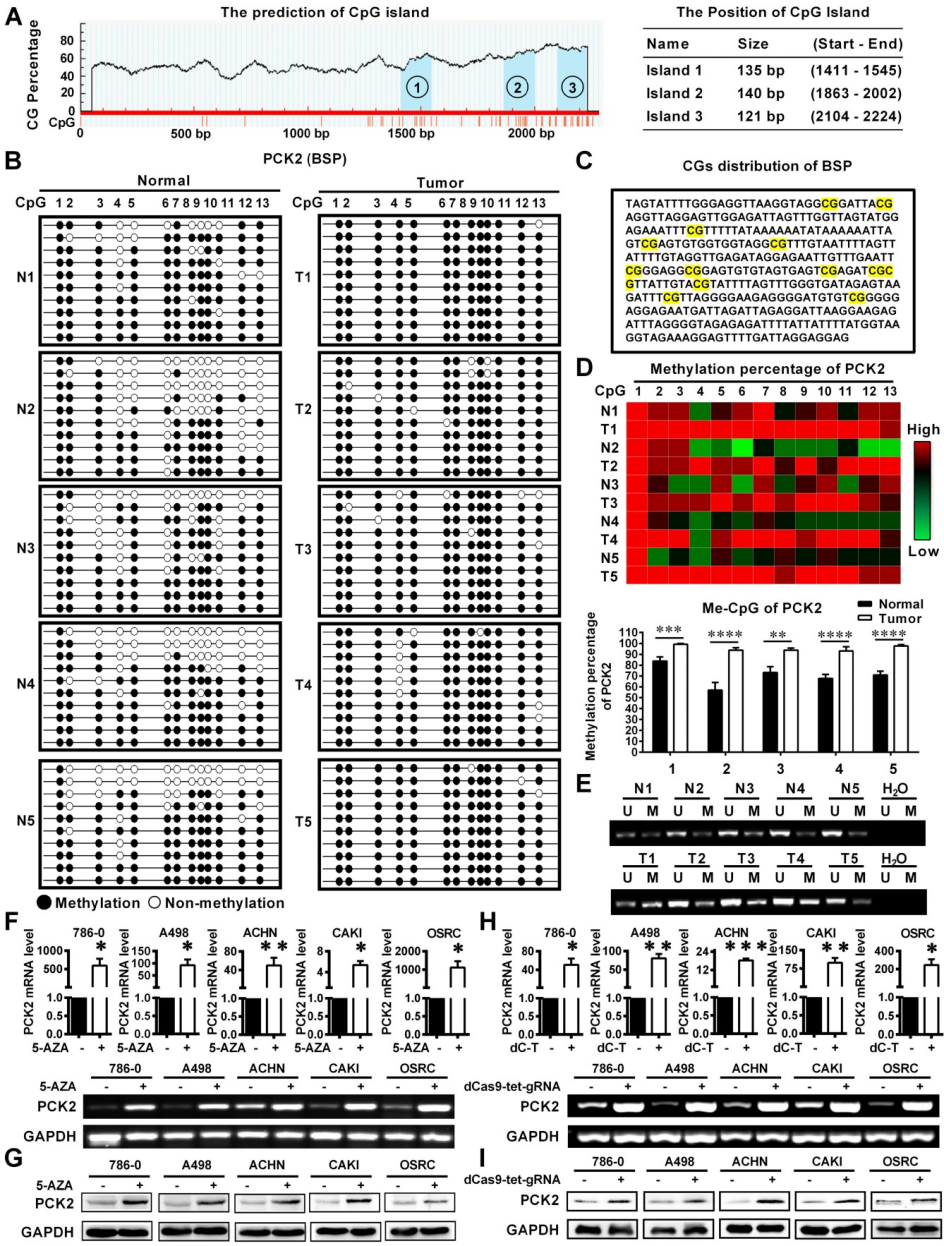
** Low expression of PCK2 in RCC is caused by DNA methylation.** (A) Prediction analysis of CpG islands in the sequence range of 2300 bp upstream from the transcriptional start site in the PCK2 promoter region (http://www.urogene.org/). (B) BSP results of PCK2 methylation status in adjacent tissues (N) and RCC tissues (T). (C) CGs distribution of BSP. (D) Heat map of methylation percentage of PCK2 and histogram of quantitative results. (E) Representative MSP results of PCK2 methylation status in adjacent tissues (N) and RCC tissues (T). (F) The mRNA levels of PCK2 in RCC cell lines after 5-AZA treatment. (G) PCK2 protein expression in RCC cell lines after 5-AZA treatment. (H) The mRNA levels of PCK2 in RCC cell lines after CRISPR/dCas9-mediated editing system specific demethylation. (I) The protein levels of PCK2 in RCC cell lines after CRISPR/dCas9-mediated editing system specific demethylation.

Based on the above results, we found a significant epigenetic difference in PCK2 between RCC and normal tissues, and this difference is responsible for the significant downregulation of PCK2 in RCC. Epigenetics, which has become an important field of biomedical research, can reveal new underlying mechanisms of biological processes 20. Therefore, the cause of epigenetic changes in PCK2 in RCC is included in the scope of this study. DNA methylation is an important cause of epigenetic changes in genes 21, 22. Through an analysis of online database, we found obvious CpG islands in the sequence range of 2300 bp upstream from the transcriptional start site in the PCK2 promoter region, which indicated that the DNA of PCK2 was likely methylated (**Figure 2A**). In order to clarify the changes of PCK2 methylation level in RCC, we performed Bisulfite Sequencing PCR (BSP) on 5 pairs of renal cell carcinoma tissues and adjacent tissues. After quantitatively detecting the methylation level, it was found that the methylation level in RCC of the PCK2 promoter region was significantly higher than its corresponding para-cancerous tissues (**Figure 2B-D**). Similarly, MSP (Methylation-Specific Polymerase Chain Reaction) experiments also confirmed that PCK2 was highly methylated in RCC (**Figure 2E, Figure S2A-B**).

In order to further explore the effect of methylation in the PCK2 promoter region on its expression in RCC, experiments on cell lines were performed. Firstly, Bisulfite Sequencing PCR (BSP) was also carried out to detect the methylation level of PCK2 in 5 different kinds of RCC cell lines. The quantitative results showed that their methylation percentages were all over 90%, which meant that they were all in a highly methylated state (**Figure S2C-F**). Subsequently, the demethylating drug 5-AZA was used to treat RCC cell lines to demonstrate the effect of methylation on the expression of PCK2. The results showed that 5-AZA could significantly increase the expression of PCK2 in RCC cell lines at both mRNA and protein levels (**Figure 2F-G**). In order to illustrate the above results more specifically, a CRISPR/dCas9-mediated editing system for PCK2 specific demethylation was constructed (**Figure S3A-C**). Similar results could be found, specific demethylation also significantly increased the expression levels of PCK2 in both mRNA and protein levels (**Figure 2H-I**). Based on the above results, conclusion could be drawn that the epigenetic changes in PCK2 in RCC, by which PCK2 was significantly downregulated were due to DNA methylation.

### PCK2 repressed the progression of RCC

**Figure 3:**
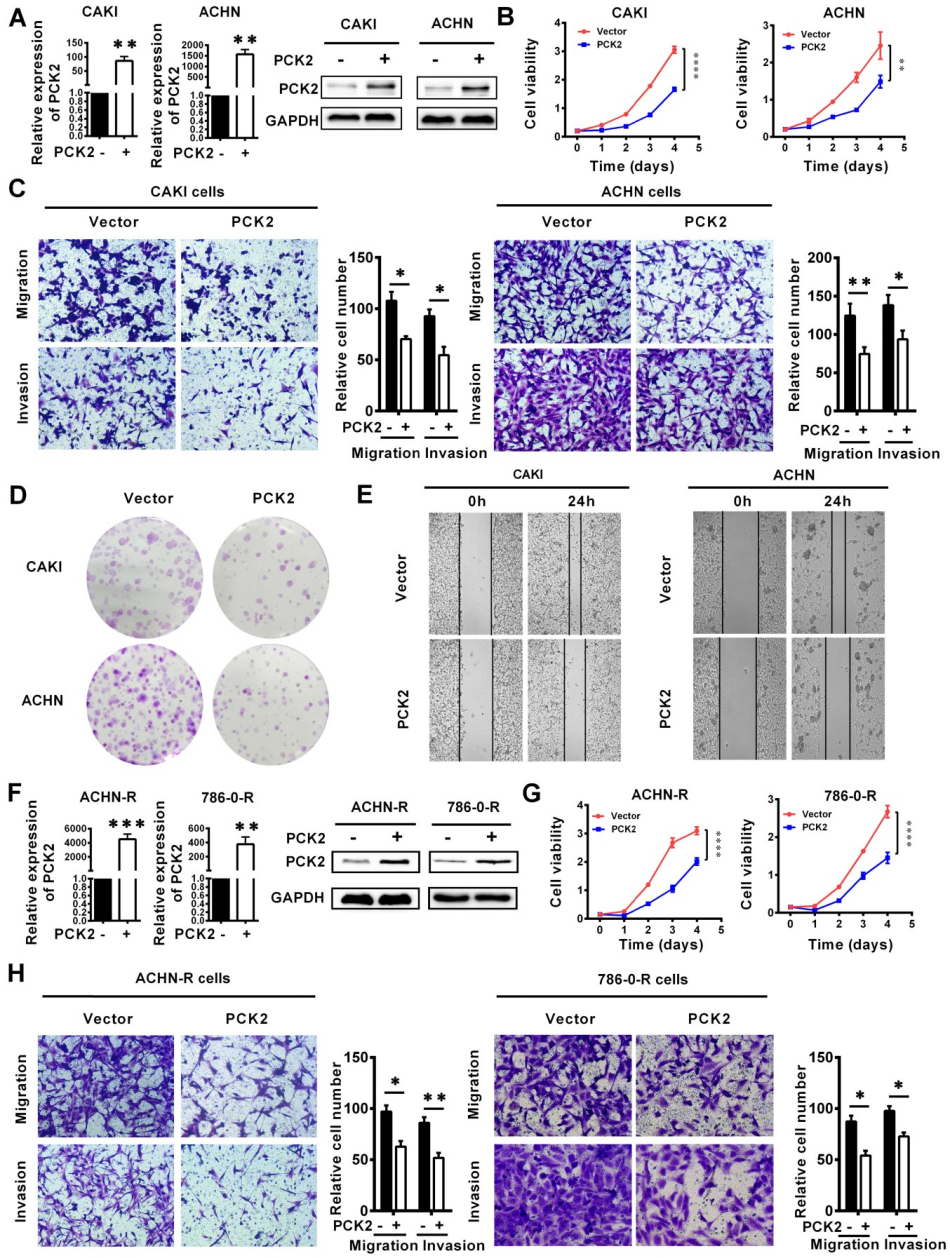
** PCK2 repressed the progression of RCC.** PCK2-overexpressing RCC cell lines were constructed by transfecting an overexpressing lentiviru. The results are plotted as the means ± SEM from three independent experiments with at least three replicates in each independent experiment. ****P < 0.0001, ***P < 0.001, **P < 0.01, and *P < 0.05. (A) Verification of PCK2 mRNA and protein overexpression levels in CAKI and ACHN cell lines. (B) Cell growth curves of CCK8 assays for CAKI and ACHN cell lines overexpressing PCK2. (C) The results of transwell assay of the migration and invasion for CAKI and ACHN cell lines overexpressing PCK2. (D) The result of colony formation assay for CAKI and ACHN cell lines overexpressing PCK2. (E) The result of wound healing assay for CAKI and ACHN cell lines overexpressing PCK2. (F) Verification of PCK2 mRNA and protein overexpression levels in ACHN-R and 786-0-R cell lines. “R” stands for sunitinib resistance. (G) Cell growth curves of CCK8 assays for ACHN-R and 786-0-R cell lines overexpressing PCK2. (H) The results of transwell assay of the migration and invasion for ACHN-R and 786-0-R cell lines overexpressing PCK2.

Based on the finding that PCK2 is greatly downregulated in RCC, we speculate that PCK2 is likely to have a certain degree of influence on the occurrence and progression of RCC. To confirm the above hypothesis, we successfully constructed CAKI and ACHN cell lines with PCK2 stably overexpressed by lentivirus (**Figure 3A**). As shown in **Figure 3B**, CCK8 proliferation curves were used to assess the proliferative capacity of the cells, and the results clearly showed that PCK2 overexpression significantly suppressed the proliferation of RCC cell lines. In addition, the migration and invasion abilities of tumors are also important characteristics to measure the degree of tumor malignancy and affect tumor progression. To measure the abilities of tumor migration and invasion, Transwell assays were carried out. Similarly, the results showed that PCK2 overexpression could significantly inhibit the migration and invasion of RCC cell lines in both CAKI and ACHN (**Figure 3C**). To more thoroughly verify the above experimental results, we subsequently performed a colony formation assay and wound healing assay. As expected, similar results were shown in **Figure 3D-E,** in which PCK2 overexpression significantly inhibited the colony formation ability and reduced the migration capacity of RCC cells.

In clinical practice, in addition to the basic types of RCC, more extensive treatments have led to the emergence of drug-resistant types of RCC. At present, these drug-resistant types of RCC should not be ignored, and among them, sunitinib-resistant RCC is the most common. To more profoundly affect clinical practice and to improve the clinical guidance of this study, we also tested the function of PCK2 in sunitinib-resistant RCC cell lines constructed in our laboratory. Similarly, we first constructed cells with stably overexpressed PCK2 in ACHN and 786-0 sunitinib resistant cell lines by lentivirus** (Figure 3F)**. As shown in **Figure 3G-H,** CCK8 and Transwell assays indicated that overexpression of PCK2 significantly inhibited the proliferation, migration and invasion of RCC sunitinib-resistant cell lines.

Subsequently, in order to further verify whether the changes in PCK2 expression caused by methylation have the same biological effects, CRISPR/dCas9-mediated editing system specifically demethylated RCC cell lines and sunitinib resistance RCC cell lines were constructed (**Figure S4A-B**). CCK8 assay showed that PCK2 expression increased by specific demethylation could still significantly inhibit RCC cell proliferation (**Figure S4C-D**). Similarly, the ability of migration and invasion could be also inhibited by the specific demethylation of PCK2 (**Figure S4E-F**). Taken together, the above experimental results allow us to conclude that PCK2 acts as a tumor suppressor to repress RCC progression.

### PCK2 significantly increased the sensitivity of RCC to sunitinib

**Figure 4:**
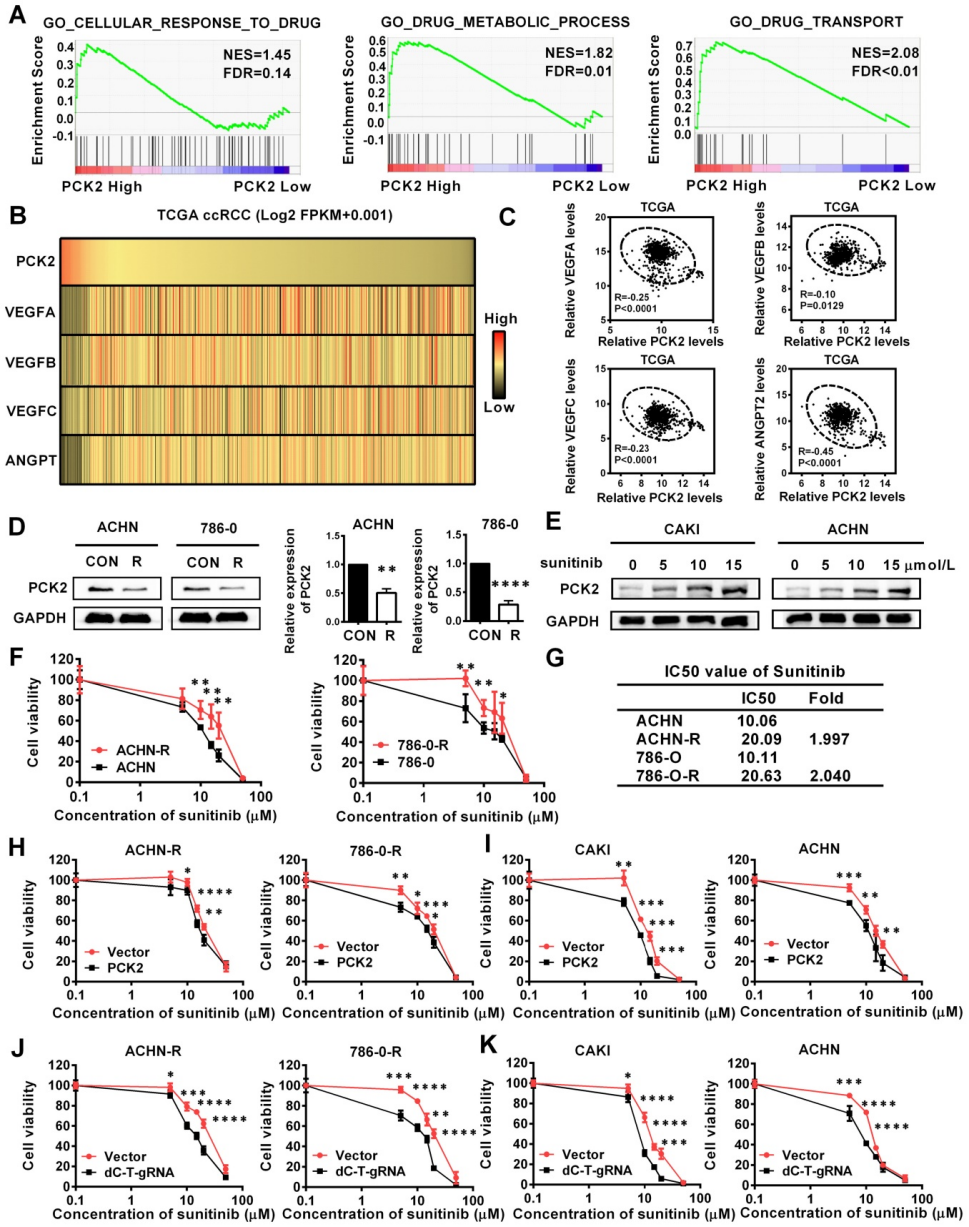
** PCK2 significantly increased the sensitivity of RCC to sunitinib.** (A) GSEA assays for the correlations between the cellular response to drug, drug metabolism and drug transport in RCC with the levels of the PCK2 mRNA, according to the TCGA database. FDR<25% and *P* < 0.05 were considered statistically significant. (B) Heatmap of the relationship between PCK2 and the VEGF family and angiogenic factors. (C) Scatter plot of the relationship among PCK2 and the VEGF family and angiogenic factors. (D) PCK2 protein and mRNA expression in ACHN-R and 786-0-R cells. (E) PCK2 protein expression in RCC cell lines after treatment with sunitinib gradient. (F) Sunitinib resistance curves for ACHN-R and 786-0-R cell lines. (G) Statistical table of the IC50 values of sunitinib for ACHN-R and 786-0-R cell lines. (H) Sunitinib sensitivity curves for ACHN-R and 786-0-R cell lines overexpressing PCK2. (I) Sunitinib sensitivity curves for CAKI and ACHN cell lines overexpressing PCK2. (J) Sunitinib sensitivity curves for ACHN-R and 786-0-R with PCK2 specific demethylation. (K) Sunitinib sensitivity curves for CAKI and ACHN with PCK2 specific demethylation. ****P < 0.0001, ***P < 0.001, **P < 0.01, and *P < 0.05.

The above experimental results confirmed that PCK2 can largely inhibit the progression of RCC, and these characteristics are mainly focused on the inhibition of the basic characteristics of tumors. In the actual process of tumor progression, clinical treatment is an important factor that cannot be ignored, among which drug therapy is especially important. For RCC, angiogenesis has become an important process affects the progression of RCC due to the extensive loss of VHL, which leads to the activation of its downstream angiogenic target genes 23. Therefore, anti-angiogenesis therapy has become the first-line treatment for RCC, and of these treatments, sunitinib is the most representative 24. The gene set enrichment analysis (GSEA) based on the data from TCGA database indicated that PCK2 was highly correlated with cellular response to drug, drug metabolic process and drug transport in RCC (**Figure 4A**). Subsequently, a correlation analysis based on the TCGA database also showed that PCK2 was highly negatively correlated with multiple angiogenic factors and angiopoietins (**Figure 4B-C**). These results imply that PCK2 is highly likely to be associated with RCC anti-angiogenic therapy. To test the above hypothesis, we first assessed the expression of PCK2 in ACHN and 786-0 sunitinib-resistant cell lines. As shown in **Figure 4D**, the expression of PCK2 was significantly decreased in sunitinib-resistant RCC cell lines in both protein and mRNA levels. Then, we performed a dosing experiment on common RCC cell lines and found that PCK2 expression gradually increased as the sunitinib concentration increased as a short-term effect of sunitinib (**Figure 4E**). Based on the response of PCK2 expression to sunitinib and its expression differences in drug-resistant RCC cell lines, we speculated that PCK2 likely exerted a synergistic effect with sunitinib in RCC. To directly show the effect of PCK2 on the drug sensitivity of RCC cell lines, we performed CCK8 assay to construct drug sensitivity curves for RCC cell lines against sunitinib. First, we constructed drug sensitivity curves of ACHN and 786-0 drug-resistant cell lines to verify the efficiency of drug resistance. The results showed that the IC50 of sunitinib in drug-resistant cells was greatly higher than control cells and exceeded 2-fold, which meant that the construction of drug-resistant cell lines was successful (**Figure 4F-G**). Subsequently, we examined the effects of PCK2 on sunitinib-resistant RCC cell lines and normal RCC cell lines on the sensitivity of sunitinib, respectively. As shown in **Figure 4H-I**, the drug sensitivity curve obtained in the CCK8 assay revealed a faster rate of decline in the PCK2 overexpression group, regardless if it is in resistant RCC cells or normal RCC cells.

In order to verify whether the specific demethylation of PCK2 had the same effect, drug sensitivity experiments were carried out in specific demethylated RCC cell lines and sunitinib-resistant cell lines. The results showed that increasing the expression of PCK2 through specific demethylation could also increase the sensitivity of cells to sunitinib (**Figure 4J-K**). From these data, we can conclude that PCK2 exerts synergistic toxicity with sunitinib in RCC and can increase the sensitivity of RCC cells to sunitinib.

### PCK2 promoted endoplasmic reticulum stress in RCC

**Figure 5:**
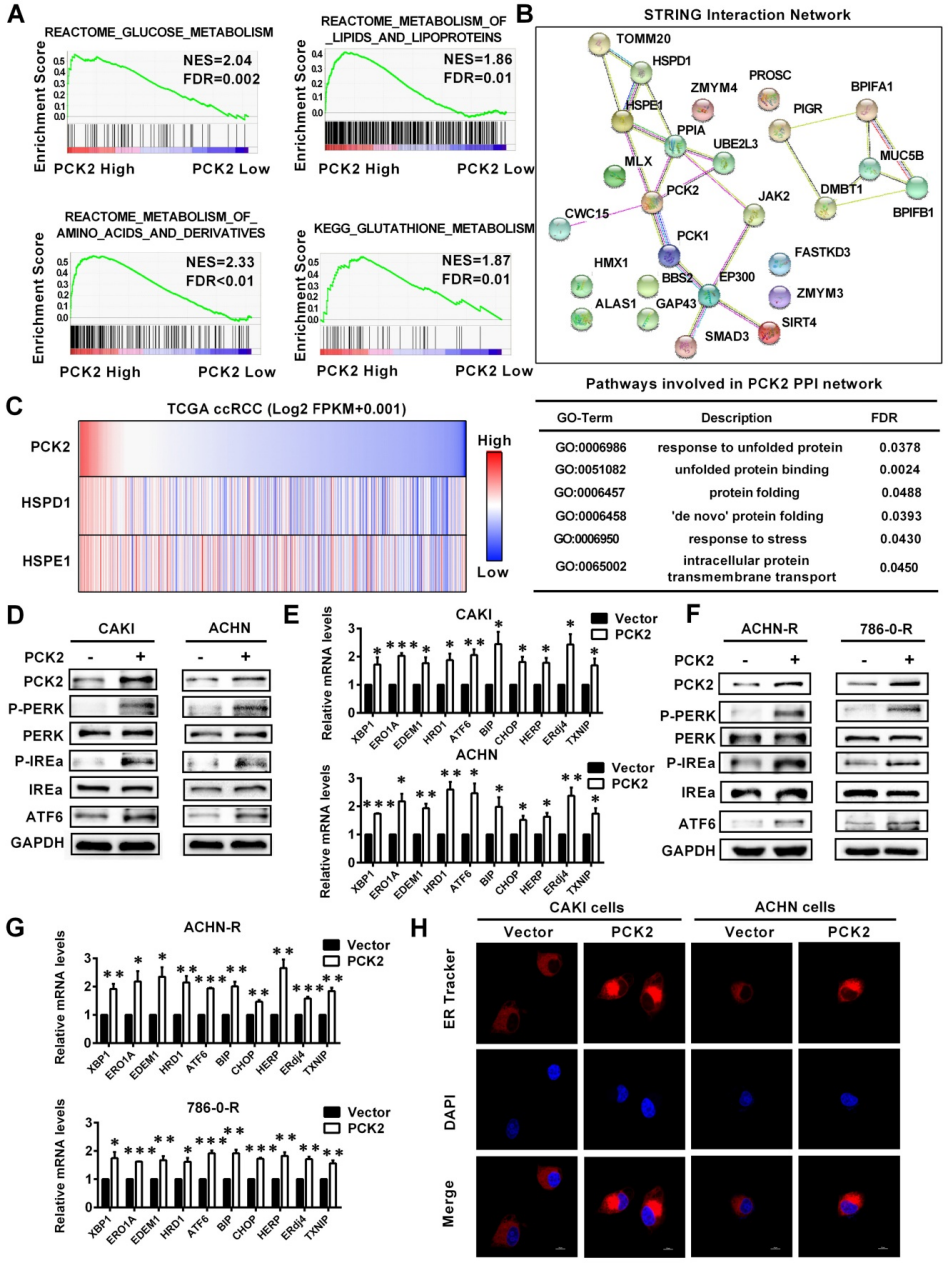
** PCK2 promoted endoplasmic reticulum stress in RCC.** (A) GSEA assays for the correlations between the glucose, lipid and amino acid metabolism in RCC with the levels of the PCK2 mRNA, according to the TCGA database. FDR<25% and P < 0.05 were considered statistically significant. (B) String interaction network and pathways involved in PCK2 PPI network. (C) Heatmap of PCK2 and unfolded protein response-related genes. (D) Protein levels of endoplasmic reticulum stress sensors in CAKI and ACHN cell lines overexpressing PCK2. (E) mRNA levels of endoplasmic reticulum stress-related genes in CAKI and ACHN cell lines overexpressing PCK2. (F) Protein levels of endoplasmic reticulum stress sensors in ACHN-R and 786-0-R cell lines overexpressing PCK2. (G) mRNA levels of endoplasmic reticulum stress-related genes in ACHN-R and 786-0-R cell lines overexpressing PCK2. (H) Immunofluorescence technology traces endoplasmic reticulum in RCC cells overexpressing PCK2, Red: ER-tracker, Blue: DAPI.

Based on the finding that PCK2 has a significant impact on biological functions of RCC, its specific mechanism and biological effects are worthy of further discussion and research. The GSEA gene enrichment analysis, which was based on the TCGA database, showed that PCK2 is highly correlated with the three basal metabolisms in RCC, including glucose metabolism, lipid metabolism, amino acid metabolism and glutathione metabolism (**Figure 5A**). It is well known that the ordering of the three basic basal metabolisms maintains the normal physiological activities of cells. Once this balance is destroyed, homeostasis in the intracellular environment will become disordered 25. The endoplasmic reticulum is widely involved in the metabolism of glucose, lipids and proteins, which plays an important role in maintaining homeostasis of the intracellular environment 26. When the physiological balance of the cell is destroyed, the endoplasmic reticulum will produce a response termed endoplasmic reticulum stress to correct the disorder of the intracellular environment 27. Considering the importance of endoplasmic reticulum stress in tumors, it was included in the scope of this study. Endoplasmic reticulum stress is induced by accumulation of unfolded or misfolded proteins, and the corresponding response effect is called unfolded protein response (UPR) 28. String interaction network analysis showed that PCK2 was closely related to protein folding and unfolded protein response related genes, and the corresponding Go-related pathways also suggested that it was closely related to unfolded protein response (**Figure 5B**). Similarly, data analysis based on TCGA also indicated that PCK2 was positively correlated with unfolded protein response related proteins (**Figure 5C**). Subsequently, we examined the expression of endoplasmic reticulum stress sensors in RCC cell lines, and the results showed that these sensors of endoplasmic reticulum stress were greatly increased in RCC cell lines with stably overexpressed PCK2 (**Figure 5D-E**). Similarly, as shown in **Figure 5F**-**G,** endoplasmic reticulum stress sensors were also greatly increased in the sunitinib-resistant RCC cell lines with stably overexpressed PCK2. Furthermore, ER Tracker imaging was conducted to visualize structural changes in the endoplasmic reticulum, and the results showed that ER tracker staining was significantly enhanced in RCC cell lines with stably overexpressed PCK2 (**Figure 5H**).

To confirm whether PCK2 specific demethylation had the same biological effect, we tested the level of endoplasmic reticulum stress sensors in RCC cell lines with CRISPR/dCas9-mediated specific demethylation. The results showed that the endoplasmic reticulum stress sensors were also significantly improved in these cell lines, which meant that the endoplasmic reticulum stress was also activated (**Figure S5A-B**). The above results indicate that PCK2 can promote endoplasmic reticulum stress in RCC.

### PCK2 suppressed the progression of RCC by promoting endoplasmic reticulum stress

**Figure 6:**
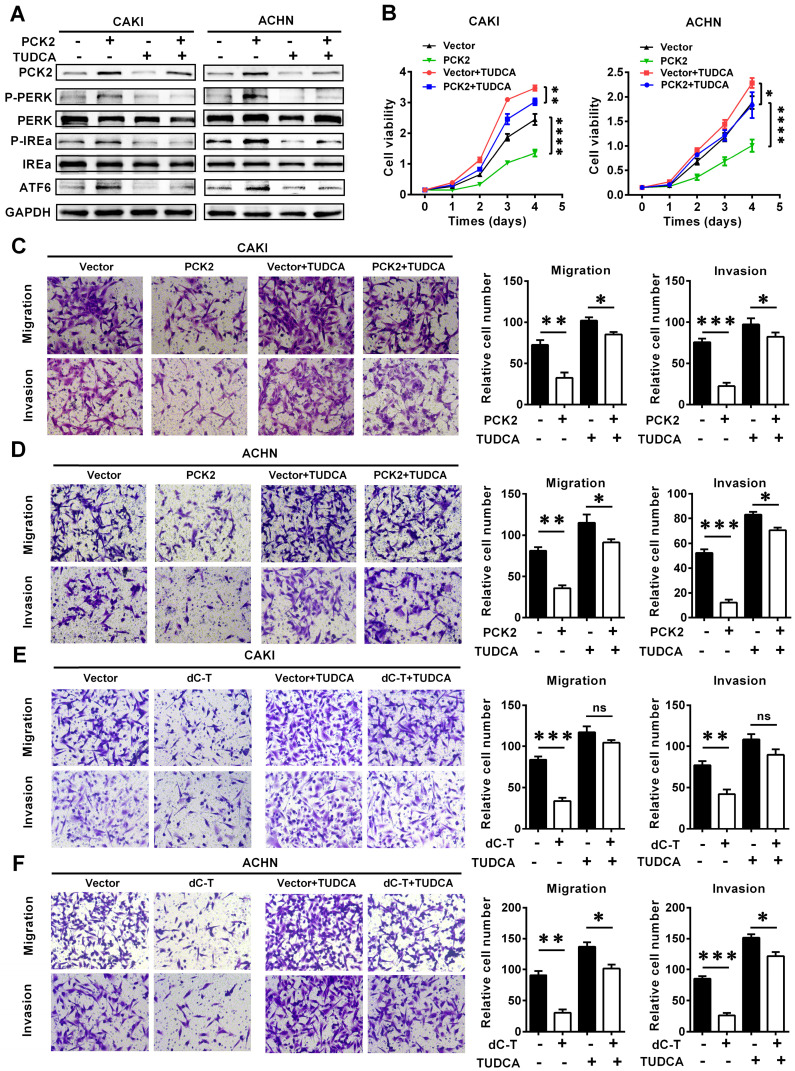
** PCK2 suppressed the progression of RCC by promoting endoplasmic reticulum stress.** Cell lines were constructed with endoplasmic reticulum stress suppressed by using the endoplasmic reticulum stress-specific inhibitor TUDCA in cell lines overexpressing PCK2 and the corresponding control cell lines to conduct functional rescue experiments. (A) Protein levels of endoplasmic reticulum stress sensors in indicated cell lines. (B) Cell growth curves of CCK8 assays for indicated cells. ****, *P* < 0.0001, **, *P* < 0.01, and *, *P* < 0.05. (C, D) Migration and invasion assay for indicated RCC cells overexpressing PCK2. (E, F) Migration and invasion assay for indicated RCC cells with PCK2 specific demethylation.

To further illustrate the role of endoplasmic reticulum stress in the PCK2-mediated biological functions in RCC, functional rescue experiments were conducted. We constructed cell lines in which endoplasmic reticulum stress was suppressed by using the endoplasmic reticulum stress-specific inhibitor TUDCA in cell lines with PCK2 overexpression and in the corresponding control cell lines (**Figure 6A**). As shown in **Figure 6B,** inhibition of endoplasmic reticulum stress could reverse the decline in cell proliferative capacity caused by overexpression of PCK2 in RCC. As for the migration and invasion ability of tumor cells, similar findings could be seen in **Figure 6C-D**, in which the inhibition of endoplasmic reticulum stress significantly attenuated the weakened migration and invasion capacities of tumor cells induced by PCK2.

In order to further verify whether PCK2 specific demethylated cell lines had the same biological effects, functional rescue experiments were carried out in these cell lines. The results showed that inhibition of endoplasmic reticulum stress could also alleviate the inhibition of RCC proliferation, migration and invasion caused by PCK2 specific demethylation (**Figure 6E-F, Figure S6A-B**). In summary, conclusion could be drawn that endoplasmic reticulum stress played an important role in the inhibition of tumor progression caused by PCK2.

### PCK2 repressed the progression of RCC *in vivo*

**Figure 7:**
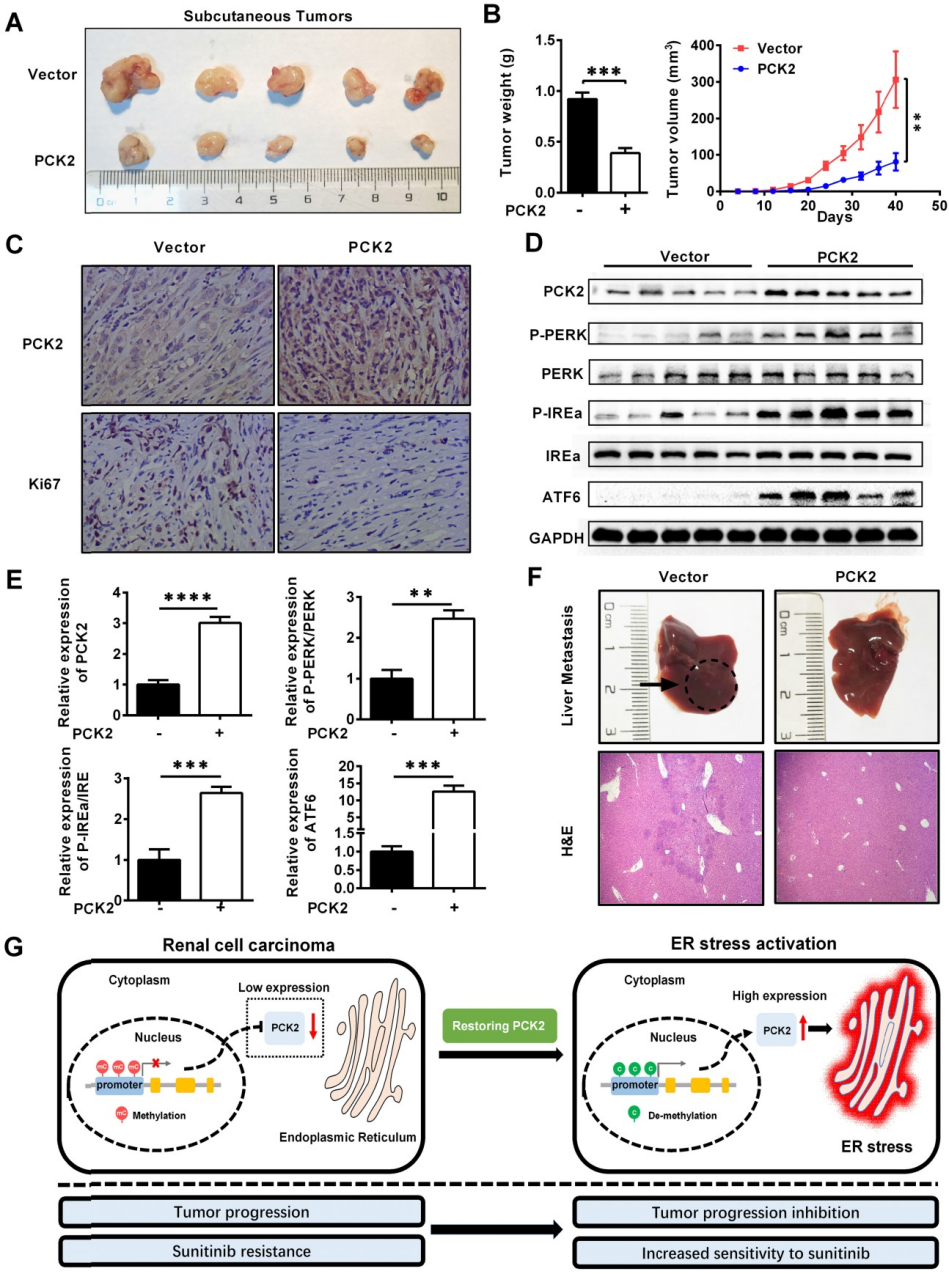
** PCK2 repressed the progression of RCC *in vivo.***(A, B) CAKI cells stably overexpressing PCK2 were injected into nude mice. Tumor size was measured every 4 days. The data are shown as the mean ± SEM for separate tumors IN each group. Images of tumors dissected from the mice. The tumor size (mm^3^) was plotted against the number of days after tumor cell implantation. Tumors were weighted after resection at the end of experiment. (C) Immunohistochemical (IHC) staining for PCK2 and the marker of tumor malignancy (KI67) in the tumor xenografts. (D, E) The levels of endoplasmic reticulum stress sensors in the tumor xenografts. (F) H&E staining of the livers in the PCK2-overexpression and control groups. (G) A model in which methylation of the PCK2 promoter region in RCC inhibits the expression of PCK2, and thus reduces endoplasmic reticulum stress resulting in the tumor progression. Restoration of PCK2 expression can activate endoplasmic reticulum stress in RCC, thereby inhibiting tumor progression and increasing tumor sensitivity to sunitinib.

Based on the results of the above cell experiments, we further evaluated the role of PCK2 in RCC *in vivo*. First, a xenograft tumor model was constructed by subcutaneous injection of CAKI cells with PCK2 overexpressed in the axilla of nude mice. As shown in **Figure 7A-B**, the volume and weight of the subcutaneous xenografts in the PCK2 overexpression group were significantly lower than those in the control group. Based on the finding that PCK2 inhibits tumor progression by activating endoplasmic reticulum stress in RCC cells, we used immunohistochemistry (IHC) and western blot to assess the level of endoplasmic reticulum stress *in vivo* and to evaluate the malignancy of the subcutaneous tumors formed. The results showed that the levels of endoplasmic reticulum stress markers were greatly increased in the PCK2 overexpression group, while the marker of tumor malignancy, KI67, was decreased in the PCK2 overexpression group (**Figure 7C-E**). Subsequently, a tail vein metastasis model in nude mice was used to assess tumor metastatic capacity *in vivo*. As shown in **Figure 7F**, a similar conclusion could be drawn that overexpression of PCK2 reduced the ability of tumors to metastasize *in vivo*. To clarify the impact of PCK2 specific demethylation *in vivo*, models of subcutaneous xenografts and tail vein metastases were also constructed by cell line with PCK2 specific demethylation. The results showed that PCK2 specific demethylation could also significantly inhibit the progression of RCC *in vivo* (**Figure S7A-D**).

Based on the above findings, we proposed a model in which methylation of the PCK2 promoter region in RCC inhibits the expression of PCK2, which reduced endoplasmic reticulum stress resulting in the tumor progression. Restoration of PCK2 expression could activate endoplasmic reticulum stress in RCC, thereby inhibiting tumor progression and increasing tumor sensitivity to sunitinib (**Figure 7G**).

## Discussion

Renal cell carcinoma (RCC) is associated with significant biological function abnormalities. Although research on RCC is gradually deepening, many enigmas and doubts about the biological function abnormalities in RCC still exist. By screening the public database, we found a protein named PCK2 that is differentially expressed in RCC and highly related to the biological function of RCC. Through in-depth research of PCK2, we found that PCK2 could inhibit RCC progression and increase the sensitivity of RCC to the targeted drug sunitinib. These findings can provide new targets and novel drug combinations for RCC treatment.

The endoplasmic reticulum is an important organelle which functions primarily in protein metabolism, while maintaining a comprehensive balance of glucose, lipids and intracellular ions in the cell 29, 30. Disruption of its function leads to an imbalance in cell homeostasis. Endoplasmic reticulum stress is a remedy to maintain cell homeostasis, and it is also one of the important mechanisms to promote cell death 14. As known, under normal physiological conditions, the maintenance of cell activity is inseparable from the function of the endoplasmic reticulum, and activation in its physiological state is one of the necessary conditions for cells to maintain normal physiological functions 31. However, the endoplasmic reticulum is extremely sensitive to stress. Under the stimulation of internal and external environmental stress factors such as hypoxia, lack of glucose, ATP depletion, calcium overload and weakened protein degradation, the endoplasmic reticulum will lose its homeostasis 28. At this time, the endoplasmic reticulum will lose the activation state under the physiological state and enter a stress state activation, which is the endoplasmic reticulum stress 32, 33. ER stress can be regarded as a special kind of endoplasmic reticulum activation. Its original intention is to try to restore the homeostasis of the endoplasmic reticulum. However, in more cases, the intracellular homeostasis is seriously damaged and difficult to correct, especially in tumors. In this case it will cause cell death, which is the famous endoplasmic reticulum stress-mediated apoptosis 34-36. Endoplasmic reticulum stress plays an important role in RCC, and it has been shown that under normal conditions, RCC can stabilize the endoplasmic reticulum as a result of its abnormal accumulation of lipids, which reduces endoplasmic reticulum stress and promotes tumor progression 15. PCK2 is highly related to metabolism, and its primary functions are to mediate energy metabolism of glucose, and maintain the homeostasis of cell by mediating the balance of comprehensive metabolism in the cell 37. Previous studies rarely involved the relationship between PCK2 and endoplasmic reticulum stress. There is only one literature report that activating endoplasmic reticulum stress in some breast cancer and colon cancer cell lines can affect the expression of PCK2 to a certain extent 38. However, there is no report about PCK2 regulating endoplasmic reticulum stress. Our study found that PCK2 can activate endoplasmic reticulum stress to inhibit the progression of RCC. This also means that it is the first time that our research has revealed that PCK2 can promote endoplasmic reticulum stress. Therefore, PCK2 can serve as a new target for RCC treatment, since it can increase the level of tumor endoplasmic reticulum stress to inhibit tumor progression.

The process of genetic information from DNA to protein is called gene expression. Due to differences in tissues, cell functions and environmental stimuli, differences in gene expression will occur under the same large genetic background, which is called the regulation of gene expression. Generally, the regulation of gene expression includes four aspects, namely, pre-transcriptional modification, transcriptional modification, post-transcriptional modification and modification of translation level. Normally, for eukaryotes, pre-translational modification has become the main way of regulating gene expression due to the particularity of their DNA structure 39-41. For PCK2, previous studies have confirmed that the mRNA level of PCK2 expression is consistent with the protein level, which means that it lacks translation and post-transcriptional modifications 7, 42-45. Therefore, when studying the expression differences of PCK2 in RCC, we pay more attention to the modification of transcription and pre-transcription level. In the epigenetic study of tumors, DNA methylation is the most important pre-translational regulatory mechanism, which can regulate gene expression by directly regulating gene transcription 46, 47. Considering that PCK2 lacks the characteristics of translation and post-transcriptional modification, methylation has become a target mechanism for studying the epigenetic differences of PCK2 in RCC.

Methylation has a certain foundation in the research of RCC. Previous studies have reported that methylation of some genes can affect the occurrence and development of RCC. The promoter of OVOLl is methylated in 40% of RCC, which can increase the expression of c-Myc, activate a variety of oncogenes, and promote the occurrence of RCC 48. SMAD6 of the TGF-B signaling pathway has also been found to be hypermethylated in RCC, which can activate the TGF-B pathway and promote tumor proliferation 49. Methylation of corticotropin releasing hormone-binding protein (CRHBP) can promote the metastasis of renal cell carcinoma, suggesting that adrenal axis hormone may play a role in the progression of RCC 50. In 44% of RCC, CHD5 is silent due to promoter methylation, which makes tumors easy to migrate and invade 51. The promoters of NELL1 and NELL2 are methylated in RCC, which makes their protein expression lower than normal kidney tissue, thereby promoting the progression of RCC 52. Apoptosis-associated speck-like protein containing CARD (ASC) is also hypermethylated in RCC. Demethylation drugs can increase its expression, thereby inhibiting the progression of RCC and activating P53 53. In addition, Bone morphogenetic protein 2 (BMP) is in a methylated state in RCC. Up-regulating the expression of BMP2 can inhibit the invasion and migration of RCC and increase apoptosis 54. PCK2 is a new tumor suppressor in RCC that we discovered, and for the first time revealed its hypermethylation status in RCC. Restoring the expression of PCK2 in RCC by specific demethylation can promote endoplasmic reticulum stress, thereby inhibiting the progression of RCC, and enhancing the sensitivity of RCC to sunitinib. This is a new discovery that is expected to provide a new mechanism and research direction for the overall research of RCC methylation.

Sunitinib is a first-line targeted drug for RCC treatment, and its use has greatly improved the prognosis of patients with RCC 3. However, with the use of sunitinib, its drawbacks have gradually emerged. In the clinical treatment process, the drug treatment efficiency often decreases, which results in insensitivity to the drug and even resistance in patients 55, 56. These are the significant challenges and difficulties faced in the clinical treatment of RCC. Ways in which to improve the sensitivity of RCC to sunitinib have also become an important topic in RCC research, as drug resistance is a problem that requires urgent resolution. Our study found that PCK2 can increase the sensitivity of common RCC cells and drug-resistant RCC cells to sunitinib. Therefore, a treatment method involving PCK2 is highly likely to provide a new combination treatment scheme for the clinical targeted therapy of RCC, thereby improving the treatment efficiency of RCC.

In addition, our study also found significant epigenetic differences in PCK2 in RCC which are manifested as significantly low expression due to high methylation of its promoter region. Combined with the finding that PCK2 inhibits the progression of RCC and increases the sensitivity to sunitinib, we can use specific drugs to reduce the methylation level of the PCK2 promoter region, thereby restoring epigenetically silenced PCK2 expression to inhibit RCC progression. This also provides a potential new direction for RCC drug development.

## Conclusions

Here, we report a marker gene that is greatly differentially expressed between RCC and normal tissues and that functionally inhibits the progression of RCC and increases the sensitivity of RCC to sunitinib by activating endoplasmic reticulum stress. It is highly likely that this gene may serve as a new target and that novel combined treatment options for RCC treatment will be developed.

## Supplementary Material

Supplementary figures and tables.

## Figures and Tables

**Table A:** List of primers

Primer	Sequence
**GADPH**	
Forward	5'-GAGTCAACGGATTTGGTCGT-3'
Reverse	5'-GACAAGCTTCCCGTTCTCAG-3'
**PCK2**	
Forward	5'-TCCCACTGGCATTCGAGATT-3'
Reverse	5'-CCGTCTTGCTCTCTACTCGT-3'
**XBP1**	
Forward	5'-AGCTCAGACTGCCAGAGATC-3'
Reverse	5'-TCACTTCATTCCCCTTGGCT-3'
**ERO1A**	
Forward	5'-CTGAACGACTTGGAGCAGTG-3'
Reverse	5'-TGTAACCAGTGTAGCGCTCA-3'
**EDEM1**	
Forward	5'-TGGAAACGATATGGTGCCCT-3'
Reverse	5'-TCTCCATCCGGTCTTCTGTG-3'
**HRD1**	
Forward	5'-ATCCTGATGACGATGGTGCT-3'
Reverse	5'-TGAAGGCCATGTACAGCAGA-3'
**ATF6**	
Forward	5'-GTGTCAGAGAACCAGAGGCT-3'
Reverse	5'-GGTGCCTCCTTTGATTTGCA-3'
**BIP**	
Forward	5'-AGGACAAGAAGGAGGACGTG-3'
Reverse	5'-ATCAGACGTTCCCCTTCAGG-3'
**CHOP**	
Forward	5'-CATTGCCTTTCTCCTTCGGG-3'
Reverse	5'-CCAGAGAAGCAGGGTCAAGA-3'
**HERP**	
Forward	5'-GAAAACCAGCCTGCCAATCA-3'
Reverse	5'-GTACATAACAACGGTGGCCC-3'
**HERP**	
Forward	5'-GAAAACCAGCCTGCCAATCA-3'
Reverse	5'-GTACATAACAACGGTGGCCC-3'
**ERDj4**	
Forward	5'-ATTTCCAGACACGCCAGGAT-3'
Reverse	5'-TCGTTGAGTGACAGTCCTGC-3'
**TXNIP**	
Forward	5'-GATACCCCAGAAGCTCCTCC-3'
Reverse	5'-ATAAGTCGGTGGTGGCATGA-3'

**Table 1:** Univariate and multivariate analyses of PCK2 mRNA level and patient overall survival (OS)

Variable	Univariate analysis			Multivariate analysis^c^		
Overall survival (n = 517)	HR^a^	95% CI^b^	*P*	HR	95% CI	*P*
**Age**						
≤60 (n = 258)	1.742	1.281-2.367	0.000*	1.706	1.252-2.325	0.001*
>60 (n = 259)						
**Gender**						
Female (n = 180)	0.956	0.701-1.303	0.774			
Male (n = 337)						
**T stage**						
T1 or T2 (n = 330)	3.038	2.243-4.114	0.000*	1.568	1.092-2.250	0.015*
T3 or T4 (n = 187)						
**N stage**						
N0 or NX (n = 503)	3.554	1.872-6.747	0.000*	2.345	1.222-4.501	0.010*
N1 (n = 14)						
**M stage**						
M0 or MX (n = 439)	4.290	3.145-5.854	0.000*	2.792	1.954-3.991	0.000*
M1 (n = 78)						
**G grade**						
G1 or G2 (n = 238)	2.607	1.855-3.662	0.000*	1.661	1.149-2.402	0.007*
G3 or G4 (n = 279)						
**PCK2**						
Low (n = 258)	0.609	0.450-0.824	0.001*	0.649	0.478-0.880	0.005*
High (n = 259)						

a, Hazard ratio, estimated from Cox proportional hazard regression model;b, Confidence interval of the estimated HR;c, Multivariate models were adjusted for T, N, M classification, age and gender.

## Abbreviations

RCC: renal cell carcinoma
PCK2: phosphoenolpyruvate carboxykinase 2
TCGA: the cancer genome atlas
ER stress: endoplasmic reticulum stress
KIRC: kidney renal clear cell carcinoma
ROC: receiver operating characteristic
AUC: area under the curve
OS: overall survival
CCK8: Cell Counting Kit-8
GSEA: gene set enrichment analysis
IHC: immunohistochemistry
T: Tumor
N: Lymph Node
M: Metastasis
G: Grade
HR: Hazard ratio
CI: Confidence interval
