# Supplementary material for: Restoring the epigenetically silenced PCK2 suppresses renal cell carcinoma progression and increases sensitivity to sunitinib by promoting endoplasmic reticulum stress
Source: Theranostics. 2020 Sep 15;10(25):11444–61. doi: 10.7150/thno.48469 (PMC7546001; doi:10.7150/thno.48469)
Supplement: Supplementary file 1 — Supplementary figures and tables. [file thnov10p11444s1.pdf]

Supplementary Table 1 Gene details of mitochondrial-related gene set ①

Differential expression of mitochondrial related genes in renal cell carcinoma													
1)	FGR	41)	P2RX7	81)	TFCP2L1	121)	MAP1B	161)	SYBU	201)	MICALL2	241)	SHMT2
2)	SLC25A5	42)	PEBP1	82)	MRPL19	122)	CKMT2	162)	AK3	202)	DEPP1	242)	GLRX5
3)	MCUB	43)	TREM2	83)	GLS	123)	PDHA1	163)	CDKN2A	203)	IFI27	243)	ABAT
4)	ACSM3	44)	ACOT7	84)	EFHD1	124)	MCCC2	164)	AUH	204)	LDHD	244)	SLC25A10
5)	AASS	45)	BIK	85)	RHOU	125)	XAF1	165)	SLC25A25	205)	GLYATL1	245)	COA3
6)	UQCRC1	46)	ACO2	86)	AGMAT	126)	RIDA	166)	GLYAT	206)	ACSF2	246)	TMEM173
7)	CHDH	47)	GZMB	87)	KMO	127)	DMGDH	167)	ACAD8	207)	ENDOG	247)	APOO
8)	CYP24A1	48)	GSTZ1	88)	ACADM	128)	HMGCS2	168)	SACS	208)	NOD2	248)	MAFF
9)	CPS1	49)	MTHFD1	89)	SDHB	129)	RSAD2	169)	ATP5F1A	209)	KLK6	249)	ANKRD37
10)	NDUFS1	50)	PCK2	90)	RAB29	130)	CMPK2	170)	PDK1	210)	DDIT4	250)	MAPT
11)	ALAS1	51)	ASB9	91)	HMGCL	131)	ECHDC3	171)	GJA1	211)	GLYCTK	251)	LRRK2
12)	MIPEP	52)	VWA8	92)	ABCD3	132)	SDS	172)	PRKCA	212)	PDHB	252)	NDUFA4
13)	STAP1	53)	OLFM4	93)	2-Mar	133)	COQ10A	173)	LRRK1	213)	P2RY12	253)	MAOA
14)	MYOM2	54)	CRYM	94)	ALDH6A1	134)	SUCLA2	174)	UCHL1	214)	P2RY1	254)	FHIT
15)	ADAM28	55)	PYCARD	95)	IFIT3	135)	TACO1	175)	ATP5MC3	215)	CDK1	255)	ACADSB
16)	MCUR1	56)	GCDH	96)	MTHFD1L	136)	ALDH1B1	176)	SFXN2	216)	HOXB9	256)	TRPV1
17)	MPC1	57)	CAV2	97)	CLU	137)	BPHL	177)	PPP2R2B	217)	NUDT6	257)	CASP4
18)	BCAT1	58)	HIBADH	98)	SLC25A51	138)	MGARP	178)	HK1	218)	GIMAP8	258)	ACSL5
19)	HAGH	59)	PHYH	99)	ACO1	139)	FDX1	179)	AIFM1	219)	PRKCE	259)	OGDHL
20)	EYA2	60)	MAPK10	100)	P4HA1	140)	SQOR	180)	HK2	220)	GATM	260)	AKR1B10
21)	OAT	61)	PPARGC1A	101)	SARDH	141)	CYP1B1	181)	ALDH4A1	221)	BCL2	261)	HIBCH
22)	MTHFD2	62)	NDUFS8	102)	G0S2	142)	IDH1	182)	ABCG1	222)	SUCLG2	262)	ECI2
23)	ACSM2B	63)	ATP5F1B	103)	RAB38	143)	HADH	183)	RDH13	223)	AGXT	263)	GK
24)	FECH	64)	CYP27B1	104)	PCK1	144)	GABARAPL1	184)	SHC1	224)	PDP2	264)	TGM2
25)	PKM	65)	ACSS3	105)	GOT2	145)	SUOX	185)	BDH1	225)	ABLIM3	265)	SDHD
26)	REEP1	66)	ELK3	106)	CLYBL	146)	SORD	186)	FDXR	226)	PC	266)	DNAJC19
27)	RPS6KA6	67)	ALDH2	107)	CCR7	147)	AC074143.1	187)	NAGS	227)	PPM1E	267)	SYNJ2BP
28)	HACD3	68)	SOD2	108)	DLGAP5	148)	PMAIP1	188)	AK4	228)	PCCA	268)	PLIN5
29)	ACAT1	69)	ALDH5A1	109)	ECHS1	149)	SDHC	189)	OMA1	229)	UCP2	269)	HOGA1
30)	GRAMD4	70)	ACOT13	110)	YWHAH	150)	SLC27A3	190)	DISC1	230)	SUGCT	270)	MRPS6
31)	SLC25A43	71)	PERP	111)	TST	151)	DEGS1	191)	SPATA18	231)	MSRA	271)	PRODH2
32)	MCCC1	72)	EPM2A	112)	RAC2	152)	TMEM177	192)	SUCLG1	232)	BNIP3	272)	NEFL
33)	SP140	73)	CCN6	113)	IVD	153)	ABHD10	193)	AIM2	233)	TYMS		
34)	CPOX	74)	GHR	114)	BBOX1	154)	ANK2	194)	ALB	234)	CAVIN1		
35)	ARG2	75)	NNT	115)	MRPL34	155)	CBR4	195)	PPM1K	235)	GLDC		
36)	PGR	76)	AGXT2	116)	ACSBG2	156)	ATG12	196)	ABHD6	236)	ERBB4		
37)	OXCT1	77)	NR3C1	117)	CYP2E1	157)	SLC25A48	197)	MTHFD2L	237)	RNF186		
38)	NOX4	78)	OGG1	118)	ASS1	158)	MMUT	198)	SLC9B2	238)	HCLS1		
39)	PPP1R15A	79)	PCCB	119)	COX7B	159)	SLC25A37	199)	ETNPPL	239)	DHTKD1		
40)	L2HGDH	80)	TFDP2	120)	MRPS25	160)	ADHFE1	200)	ACSL6	240)	MRPL41		

Gene set ① is derived from the mitochondrion (goterm) project in the "Gene array analysis of clear cell renal cell carcinoma tissue versus matched normal kidney tissue" data subset of the EMBL-EBI database.

Supplementary Table 2 Gene details of mitochondrial-related gene set ②

Differential expression of mitochondrial related genes in renal cell carcinoma													
1)	MCUB	31)	NOX4	61)	TFDP2	91)	ASS1	121)	ABHD10	151)	GRPEL2	181)	TYMS
2)	ACSM3	32)	P2RX7	62)	TFCP2L1	92)	SESN2	122)	ANK2	152)	DEPP1	182)	CAVIN1
3)	PNPLA4	33)	HDAC6	63)	GPD2	93)	MRPS25	123)	ATG12	153)	IFI27	183)	GLDC
4)	AASS	34)	TREM2	64)	GLS	94)	MAP1B	124)	SLC25A48	154)	TMEM135	184)	ERBB4
5)	UQCRC1	35)	ACOT7	65)	EFHD1	95)	CKMT2	125)	MTFR2	155)	LDHD	185)	RNF186
6)	LARS2	36)	GCAT	66)	KYNU	96)	MCCC2	126)	ADHFE1	156)	GLYATL1	186)	TCAIM
7)	BID	37)	GZMB	67)	AGMAT	97)	XAF1	127)	AUH	157)	ACSF2	187)	HCLS1
8)	CHDH	38)	PCK2	68)	KMO	98)	RIDA	128)	SLC25A25	158)	KLK6	188)	DHTKD1
9)	CYP24A1	39)	ASB9	69)	ACADM	99)	DMGDH	129)	GLYAT	159)	DDIT4	189)	MRPL41
10)	NDUFS1	40)	VWA8	70)	SDHB	100)	HMGCS2	130)	ACAD8	160)	GLYCTK	190)	ABAT
11)	STAP1	41)	OLFM4	71)	RAB29	101)	RSAD2	131)	SACS	161)	PDHB	191)	SLC25A10
12)	MYOM2	42)	CRYM	72)	HMGCL	102)	CMPK2	132)	PDK1	162)	P2RY12	192)	APOO
13)	ADAM28	43)	PYCARD	73)	2-Mar	103)	ECHDC3	133)	GJA1	163)	P2RY1	193)	ANKRD37
14)	MCUR1	44)	CAV2	74)	ALDH6A1	104)	COQ10A	134)	PRKCA	164)	CDK1	194)	LRRK2
15)	MPC1	45)	NUDT1	75)	MTHFD1L	105)	ALDH1B1	135)	UCHL1	165)	NUDT6	195)	MAOA
16)	BCAT1	46)	PHYH	76)	CLU	106)	BPHL	136)	SFXN2	166)	GIMAP8	196)	FHIT
17)	HAGH	47)	PPARGC1A	77)	SLC25A51	107)	RNF144B	137)	PPP2R2B	167)	PRKCE	197)	ACADSB
18)	OAT	48)	NDUFS8	78)	ACO1	108)	FDX1	138)	AIFM1	168)	GATM	198)	CASP4
19)	MTHFD2	49)	CYP27B1	79)	P4HA1	109)	BCL2L10	139)	HK2	169)	BCL2	199)	ACSL5
20)	ACSM2B	50)	ACSS3	80)	G0S2	110)	CYP1B1	140)	ALDH4A1	170)	SUCLG2	200)	OGDHL
21)	FECH	51)	ALDH2	81)	RAB38	111)	IDH1	141)	ABCG1	171)	PDP2	201)	AKR1B10
22)	PKM	52)	ALDH5A1	82)	PCK1	112)	PARP9	142)	BDH1	172)	ABLIM3	202)	HIBCH
23)	REEP1	53)	ACOT13	83)	CLYBL	113)	HADH	143)	NAGS	173)	PC	203)	ECI2
24)	RPS6KA6	54)	ADGRG6	84)	DLGAP5	114)	GABARAPL1	144)	AK4	174)	PPM1E	204)	MT-ND5
25)	ACAT1	55)	EPM2A	85)	ECHS1	115)	SUOX	145)	SLC25A34	175)	PCCA	205)	GK
26)	GRAMD4	56)	THG1L	86)	YWHAH	116)	SORD	146)	SPATA18	176)	UCP2	206)	TGM2
27)	SLC25A43	57)	AGXT2	87)	TST	117)	AC074143.1	147)	SUCLG1	177)	SUGCT	207)	HAUS3
28)	MCCC1	58)	NR3C1	88)	RAC2	118)	SLC27A3	148)	ALB	178)	MSRA	208)	HOGA1
29)	CPOX	59)	OGG1	89)	IVD	119)	SFXN5	149)	PPM1K	179)	DOK7	209)	PRODH2
30)	ARG2	60)	PCCB	90)	ACSBG2	120)	TMEM177	150)	ETNPPL	180)	BDNF	210)	NEFL

Gene set ② is derived from the mitochondrion (goterm) project in the " Transcription profiling of clear cell renal carcinomas and normal kidney cortical tissues" data subset of the EMBL-EBI database.

Supplementary Table 3 Gene details of mitochondrial-related gene set ③

Differential expression of mitochondrial related genes in renal cell carcinoma			
1) AASS	31) DHRS4	61) MRPS28	91) SLC25A10
2) ACAA2	32) ECHS1	62) MUT	92) SLC25A13
3) ACAD8	33) ETFDH	63) NDUFA1	93) SLC25A15
4) ACADS	34) FDX1	64) NDUFA11	94) SLC25A16
5) ACADSB	35) FECH	65) NDUFA4	95) SLC25A20
6) ACAT1	36) FH	66) NDUFA6	96) SLC25A29
7) ACSS1	37) GATM	67) NDUFB8	97) SLC25A33
8) AGMAT	38) GCAT	68) NDUFS1	98) SLC25A35
9) AGXT2	39) GCDH	69) NDUFS2	99) SLC25A38
10) AIFM1	40) GCSH	70) NDUFS8	100) SLC25A4
11) ALDH1B1	41) GLDC	71) NNT	101) SLC25A42
12) ALDH6A1	42) GLRX5	72) NUDT9	102) SLC25A5
13) AMT	43) GLS	73) OGG1	103) SUCLG1
14) ARG2	44) GLS2	74) OXCT1	104) SUCLG2
15) ATP5D	45) GLYAT	75) PC	105) SUOX
16) ATP5G3	46) GOT2	76) PCCA	106) SYNJ2BP
17) ATP5L	47) GSTZ1	77) PCCB	107) TFAM
18) ATP5S	48) HADH	78) PCK2	108) TIMM8A
19) ATPIF1	49) IDH2	79) PDHA1	109) TOMM40
20) AUH	50) IDH3G	80) PDHB	110) TRNT1
21) BCKDHB	51) ISCU	81) PDP1	111) TSFM
22) BCL2L1	52) LDHD	82) PPA2	112) TXNRD2
23) C21orf33	53) LETM1	83) PPIF	113) UQCRB
24) CHDH	54) MAOA	84) PPP2R1B	
25) CKMT1B	55) ME3	85) PRDX3	
26) CKMT2	56) MIPEP	86) SDHB	
27) CPT2	57) MOSC2	87) SFXN1	
28) CRAT	58) MRPL34	88) SFXN2	
29) CYP11B2	59) MRPL44	89) SFXN5	
30) CYP27B1	60) MRPS25	90) SIRT3	

Gene set ③ is derived from the mitochondrion Go Cellular Component (GO) project in the " differentially expressed genes in renal cell carcinoma in Lenburg Renal " data subset of the Ocomine database.

Supplementary Table 4 Gene details of mitochondrial-related gene set ④

Differential expression of mitochondrial related genes in renal cell carcinoma			
1) ABCB9	36) COX7A2	71) ITPK1	106) PDHA1
2) ACAA2	37) COX7B	72) LARS2	107) PDHB
3) ACAD8	38) COX7C	73) MCCC2	108) PINK1
4) ACADS	39) COX8A	74) MRPL12	109) PPA2
5) ACADSB	40) CPT1A	75) MRPL28	110) PRDX3
6) ACAT1	41) CYC1	76) MRPL34	111) 4-Sep
7) AIFM1	42) CYP11B2	77) MRPL49	112) SIRT3
8) AK2	43) CYP27B1	78) MRPS31	113) SLC25A10
9) AKAP1	44) DBT	79) MRPS35	114) SLC25A15
10) ALAS1	45) DECR1	80) MTX2	115) SLC25A20
11) ALDH2	46) DHRS4	81) MUT	116) SLC25A3
12) ALDH6A1	47) DLD	82) NDUFA1	117) SLC25A38
13) AMT	48) ECHDC2	83) NDUFA10	118) SLC25A4
14) ARG2	49) ECHS1	84) NDUFA3	119) SLC25A5
15) ATP5A1	50) ENDOG	85) NDUFA4	120) SUCLA2
16) ATP5B	51) FDX1	86) NDUFA6	121) SUCLG1
17) ATP5C1	52) FECH	87) NDUFA8	122) SUCLG2
18) ATP5D	53) FH	88) NDUFB1	123) SUOX
19) ATP5E	54) FTSJ3	89) NDUFB2	124) SURF1
20) ATP5G1	55) GATM	90) NDUFB3	125) SYNJ2BP
21) ATP5G3	56) GCAT	91) NDUFB5	126) TSFM
22) ATP5J	57) GCDH	92) NDUFB8	127) TUFM
23) ATP5J2	58) GCSH	93) NDUFS1	128) TXNRD2
24) ATP5L	59) GLDC	94) NDUFS2	129) UQCRC1
25) ATP5O	60) GLRX5	95) NDUFS8	130) UQCRFS1
26) ATPIF1	61) GLS	96) NDUFV1	131) UQCRH
27) AUH	62) GLYAT	97) NFS1	
28) BCKDHB	63) GOT2	98) NIPSNAP1	
29) BCL2	64) HADH	99) NNT	
30) C21orf33	65) HMGCS2	100) OGG1	
31) CKMT2	66) HSD17B10	101) OXCT1	
32) COQ3	67) IDH2	102) OXCT2	
33) COX4I1	68) IDH3G	103) PCCA	
34) COX5A	69) IMMT	104) PCCB	
35) COX6B1	70) ISCU	105) PCK2	

Gene set ④ is derived from the mitochondrion Go Cellular Component (GO) project in the " differentially expressed genes in renal cell carcinoma in Beroukhim Renal " data subset of the Ocomine database.

Supplementary Table 5 Gene details of energy metabolism-related gene set ⑤ and ⑥

Differential expression of energy metabolism-related genes in renal cell carcinoma	
Gene set ⑤	Gene set ⑥
1) MDH1	1) NUP160
2) PFKP	2) PFKP
3) PKM	3) PKM
4) PCK2	4) PCK2
5) ALDOC	5) ALDOC
6) ENO2	6) ENO2
7) PFKFB4	7) NUP155
8) GOT1	8) PFKFB4
9) PFKFB2	9) GOT1
10) PCK1	10) PFKFB2
11) GOT2	11) PCK1
12) G6PC	12) G6PC
13) ALDOB	13) ALDOB
14) PKLR	14) PKLR
15) PFKM	15) HK2
16) HK1	16) FBP1
17) HK2	17) PGM2L1
18) FBP1	18) PFKFB3
19) PGM2L1	19) PC
20) PFKFB3	20) SLC25A10
21) PC	
22) SLC25A10	

1) Gene set ⑤ is derived from the Glucose metabolism (pathwayname) project in the "Gene array analysis of clear cell renal cell carcinoma tissue versus matched normal kidney tissue" data subset of the EMBL-EBI database.
2) Gene set ⑥ is derived from the Glucose metabolism (pathwayname) project in the " Transcription profiling of clear cell renal carcinomas and normal kidney cortical tissues" data subset of the EMBL-EBI database.

Supplementary figure legends

Supplemental Figure 1. The expression of PCK2 is highly correlated with the clinical prognosis of RCC. (A) The expression of PCK2 in four independent additional gene sets from the Oncomine database**.** (B) The Kaplan-Meier curves of overall survival based on PCK2 expression were constructed for subgroups of patients with RCC (Male; Female; Age > 60; T1+T2 stage; N0 stage; Non-metastasis; TNM I + II stage; G1+G2 stage).

Supplemental Figure 2. PCK2 is hypermethylated in RCC cell lines. (A) The CGs distribution of MSP. (B) The primers sequence of MSP. (C) BSP results of PCK2 methylation status in RCC cell lines. (D) The CGs distribution of BSP. (E) The methylation status of each CpG site in RCC cell lines. (F) The quantitative statistical histogram of the methylation percentage of PCK2 in RCC.

Supplemental Figure 3. Construction principle of CRISPR/dCas9-mediated editing system for PCK2 specific demethylation. (A) Schematic diagram of the working principle of the CRISPR/dCas9-mediated editing system for specific demethylation. (B) Schematic diagram of CRISPR/dCas9-mediated editing system for PCK2 specific demethylation. (C) Construction sequence of CRISPR/dCas9-mediated editing system for PCK2 specific demethylation.

Supplemental Figure 4. The progression of RCC is inhibited by the specific demethylation of PCK2.

PCK2 specific demethylated RCC cell lines were constructed by CRISPR/dCas9-mediated editing system. The results are plotted as the means ± SEM from three independent experiments with at least three replicates in each independent experiment. ****$P < 0.0001$, ***$P < 0.001$, **$P < 0.01$, and *$P < 0.05$. (A) Verification of PCK2 mRNA and protein levels of specific demethylation in CAKI and ACHN cell lines. (B) Verification of PCK2 mRNA and protein levels of specific demethylation in ACHN-R and 786-0-R cell lines. "R" stands for sunitinib resistance. (C) Cell growth curves of CCK8

assays for CAKI and ACHN with PCK2 specific demethylation. (D) Cell growth curves of CCK8 assays for ACHN-R and 786-0-R cell lines with PCK2 specific demethylation. (E) The results of transwell assay of the migration and invasion for CAKI and ACHN cell lines with PCK2 specific demethylation. (F) The results of transwell assay of the migration and invasion for ACHN-R and 786-0-R cell lines with PCK2 specific demethylation.

Supplemental Figure 5. The specific demethylation of PCK2 promotes endoplasmic reticulum stress in RCC. (A) Protein levels of endoplasmic reticulum stress sensors in CAKI and ACHN cell lines with PCK2 specific demethylation. (B) Protein levels of endoplasmic reticulum stress sensors in ACHN-R and 786-0-R cell lines with PCK2 specific demethylation.

Supplemental Figure 6. Endoplasmic reticulum stress relieves the inhibition of RCC proliferation caused by PCK2 specific demethylation. Cell lines were constructed with endoplasmic reticulum stress suppressed by using the endoplasmic reticulum stress-specific inhibitor TUDCA in cell lines with PCK2 specific demethylation and the corresponding control cell lines to conduct functional rescue experiments. (A, B) Cell growth curves of CCK8 assays for indicated cells. ****, $P < 0.0001$, **, $P < 0.01$, *, $P < 0.05$ and ns, no significance.

Supplemental Figure 7. PCK2 specific demethylation inhibited the progression of RCC in vivo. Models of subcutaneous xenografts and tail vein metastases were constructed by cell line with PCK2 specific demethylation. (A) Tumor volume for indicated models. (B) The weight of the tumors for indicated models. (C) The growth curve of tumers for indicated models. (D) Liver metastasis status for indicated models.

Supplemental Fig. 1

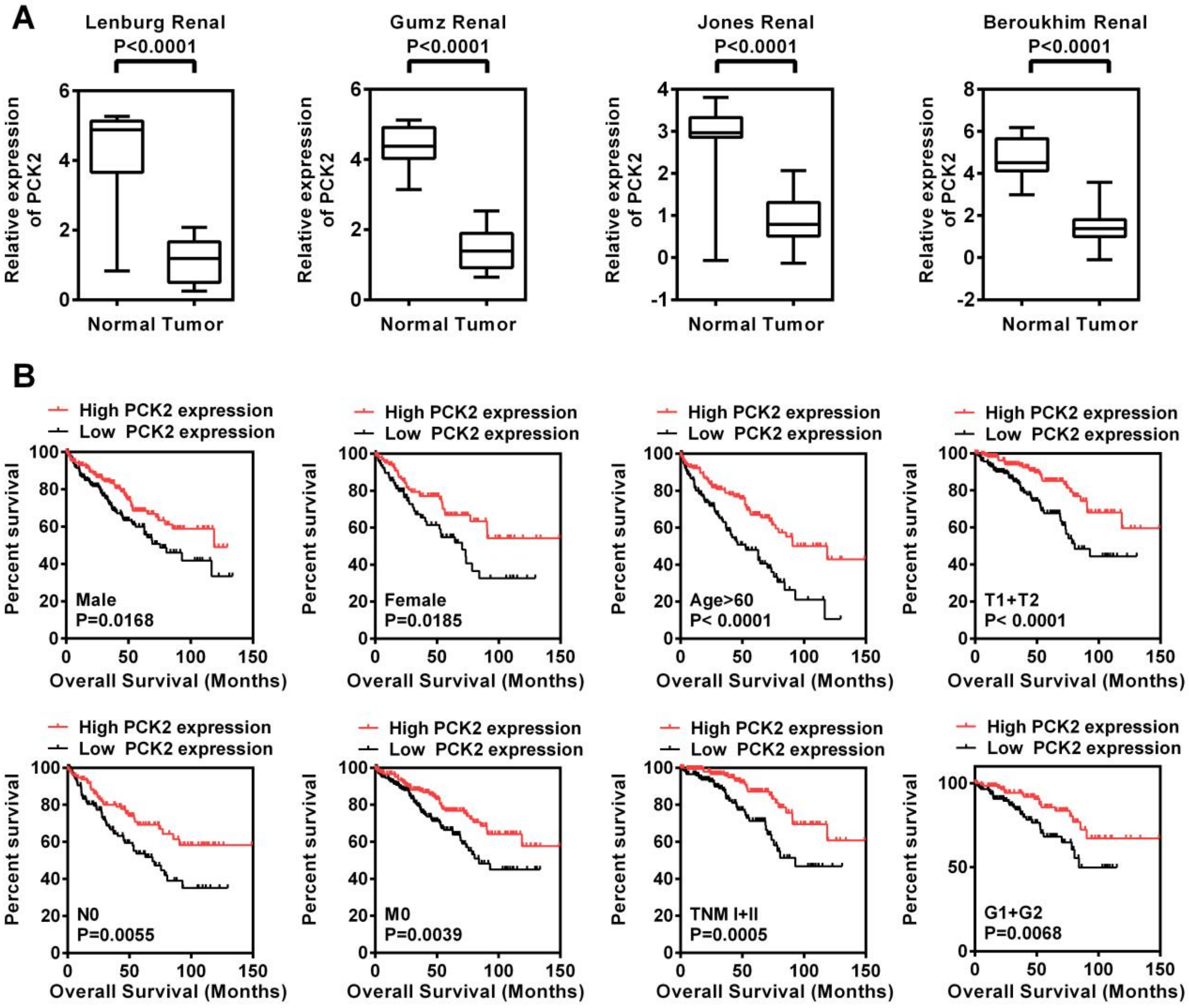

A

CGs distribution of MSP

TGTTTGGAGTTTCGGGGTCGAGGGAGT
TGGTTTGTTAGCGGGGCGGAGGAAAG
TTAGTGTTAGTTTTATTAGGTTTCGTTTT
CGCGTTTGTTTTTTTTTTTTTTTAAGCGTT
TTTCGTTAGTTTTTGTTGTGGTTCGTT

B

Primers Sequence of MSP

Name	Sequence (5-3)
H-pck2c(M)-F	GTTTGGAGTTTCGGGGTCGA
H-pck2c(M)-R	CGAAACGAACCACAACAAAAAC
H-pck2c(U)-F	GGAGTTTGGAGTTTTGGGGTTG
H-pck2c(U)-R	AACAAAACAAACCACAACAAAAACT

C

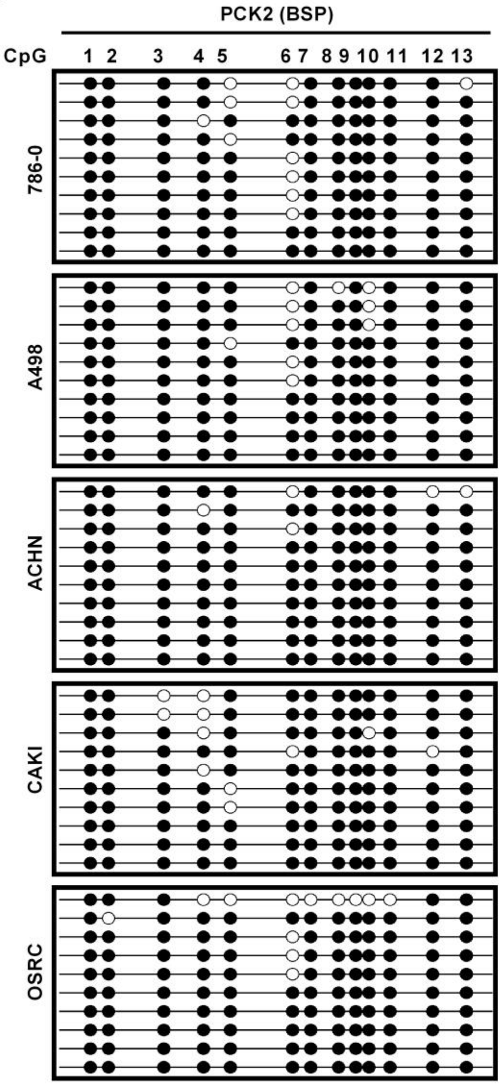

D

CGs distribution of BSP

TAGTATTTTGGGAGGTTAAGGTAGGCGGATTACGAGG
TTAGGAGTTGGAGATTAGTTTGGTTAGTATGGAGAAAT
TTCGTTTTTATAAAAAATATAAAAAATTAGTCGAGTGT
GGTGGTAGGCGTTTGTAATTTTAGTTATTTTGTAGGTTG
AGATAGGAGAATTGTTTGAATTCGGGAGGCGGAGTGT
GTAGTGAGTCGAGATCGCGTTATTGTACGTATTTTAGT
TTGGGTGATAGAGTAAGATTTCGTTAGGGGAAGAGGG
GATGTGTCGGGGGAGGAGAATGATTAGATTAGAGGAT
TAAGGAAGAGATTTAGGGGTAGAGAGATTTTATTATTT
TATGGTAAGGTAGAAAGGAGTTTTGATTAGGAGGAG

E

Methylation status of each CpG site

CpG	1	2	3	4	5	6	7	8	9	10	11	12	13	Total
786-0	100	100	100	90	70	40	100	100	100	100	100	100	90	91.5 (%)
A498	100	100	100	100	90	50	100	90	100	70	100	100	100	92.3 (%)
ACHN	100	100	100	90	100	80	100	100	100	100	100	90	90	96.2 (%)
CAKI	100	100	80	60	80	90	100	100	100	90	100	90	100	91.5(%)
OSRC	100	90	100	90	90	60	90	90	90	90	90	100	100	90.8(%)

The percentage represents the degree of methylation of CGs in PCK2

F

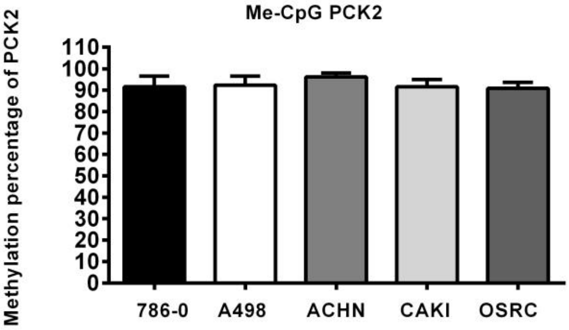

Supplemental Fig. 3

A B

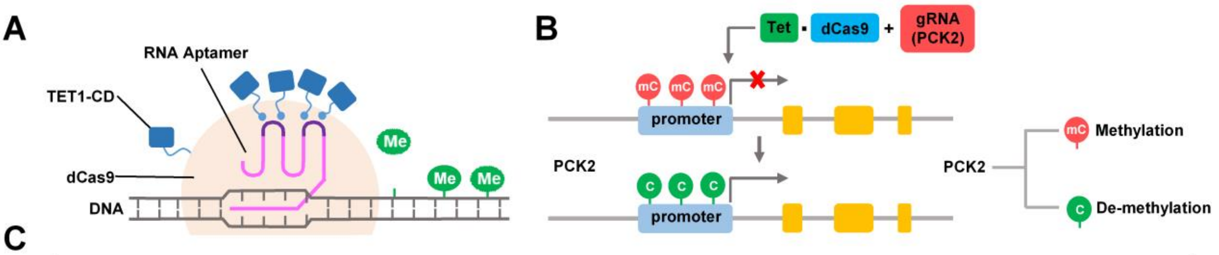

C

U6-sgRNA(PCK2)*4
GCTAGCGCTACCGGACTCAGGAGGGCCTATTTCCCATGATTCCTTCATATTTGCATATACGATACAAGGC
TGTTAGAGAGATAATTAGAATTAATTTGACTGTAAACACAAAGATATTAGTACAAAATACGTGACGTAGAAA
GTAATAATTTCTTGGGTAGTTTGCAGTTTTAAAATTATGTTTTAAAATGGACTATCATATGCTTACCGTAACT
TGAAAGTATTTCGATTTCTTGGCTTTATATATCTTGTGGAAAGGACGAAACACCGTAAGACTCCGTCAGGG
GAAGGTTTTAGAGCTAGAAATAGCAAGTTAAAATAAGGCTAGTCCGTTATCAACTTGAAAAAGTGGCACC
GAGTCGGTGCTTTTTTACCGGTGAGGGCCTATTTCCCATGATTCCTTCATATTTGCATATACGATACAAGG
CTGTTAGAGAGATAATTAGAATTAATTTGACTGTAAACACAAAGATATTAGTACAAAATACGTGACGTAGAA
AGTAATAATTTCTTGGGTAGTTTGCAGTTTTAAAATTATGTTTTAAAATGGACTATCATATGCTTACCGTAAC
TTGAAAGTATTTCGATTTCTTGGCTTTATATATCTTGTGGAAAGGACGAAACACCGGGTGGAGTCTCTCTA
CCCCTGTTTTAGAGCTAGAAATAGCAAGTTAAAATAAGGCTAGTCCGTTATCAACTTGAAAAAGTGGCACC
GAGTCGGTGCTTTTTTCTCGAGGAGGGCCTATTTCCCATGATTCCTTCATATTTGCATATACGATACAAGG
CTGTTAGAGAGATAATTGGAATTAATTTGACTGTAAACACAAAGATATTAGTACAAAATACGTGACGTAGA
AAGTAATAATTTCTTGGGTAGTTTGCAGTTTTAAAATTATGTTTTAAAATGGACTATCATATGCTTACCGTAA
CTTGAAAGTATTTCGATTTCTTGGCTTTATATATCTTGTGGAAAGGACGAAACACCGAAGTTACATCATGT
GCGGCTGTTTTAGAGCTAGAAATAGCAAGTTAAAATAAGGCTAGTCCGTTATCAACTTGAAAAAGTGGCA
CCGAGTCGGTGCTTTTTTGGATCCGAGGGCCTATTTCCCATGATTCCTTCATATTTGCATATACGATACAA
GGCTGTTAGAGAGATAATTGGAATTAATTTGACTGTAAACACAAAGATATTAGTACAAAATACGTGACGTA
GAAAGTAATAATTTCTTGGGTAGTTTGCAGTTTTAAAATTATGTTTTAAAATGGACTATCATATGCTTACCG
TAACTTGAAAGTATTTCGATTTCTTGGCTTTATATATCTTGTGGAAAGGACGAAACACCGCTGGCGGGAG
GCGCTTAAAAGTTTTAGAGCTAGAAATAGCAAGTTAAAATAAGGCTAGTCCGTTATCAACTTGAAAAAGTG
GCACCGAGTCGGTGCTTTTTTCTGCAGTCGACGGTACCGCGG

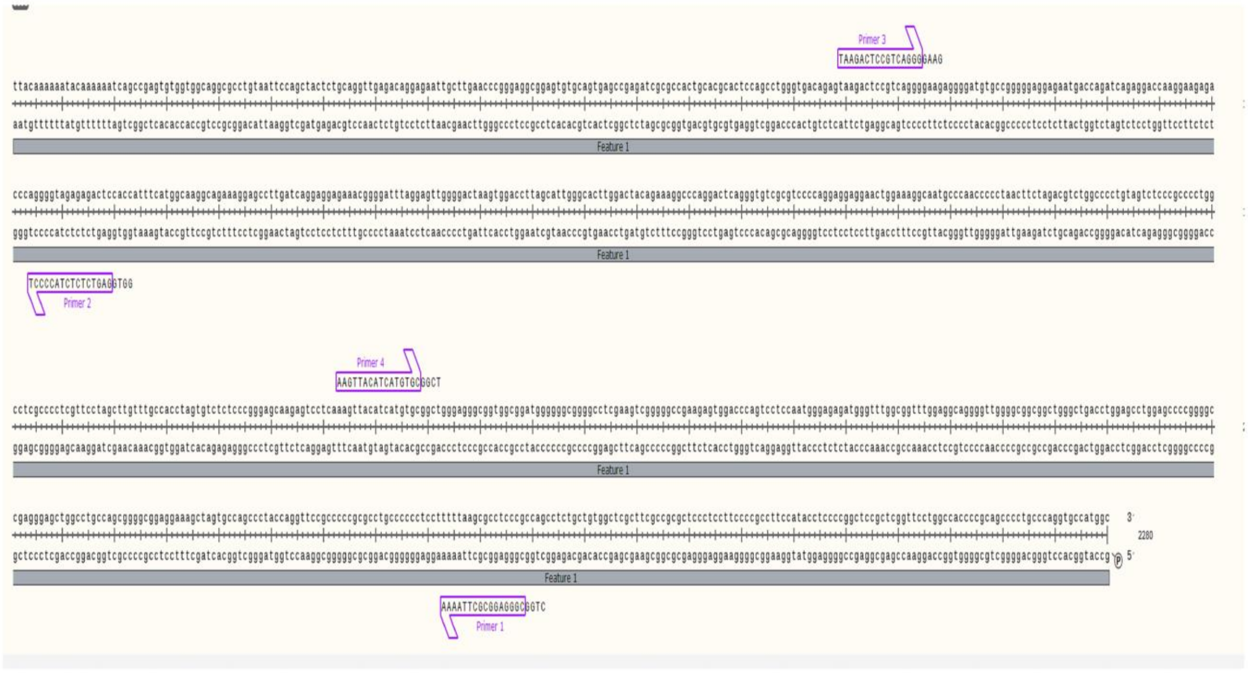

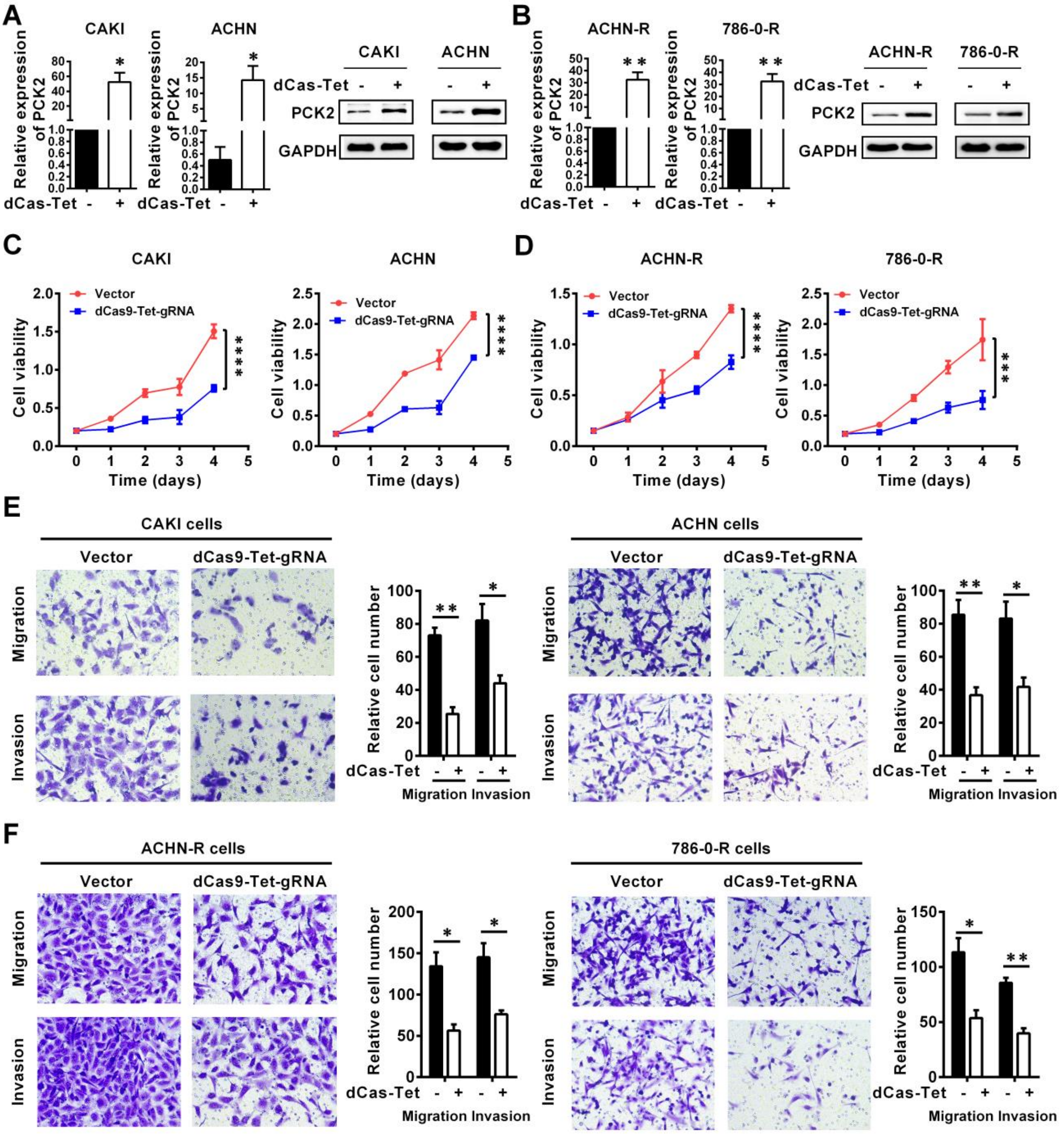
A
CAKI
ACHN
Relative expression of PCK2
dCas-Tet - +
PCK2
GAPDH
B
ACHN-R
786-0-R
C
CAKI
ACHN
Vector
dCas9-Tet-gRNA
Cell viability
Time (days)
D
ACHN-R
786-0-R
E
CAKI cells
ACHN cells
Vector
dCas9-Tet-gRNA
Migration
Invasion
Relative cell number
Migration Invasion
F
ACHN-R cells
786-0-R cells

Supplemental Fig. 5

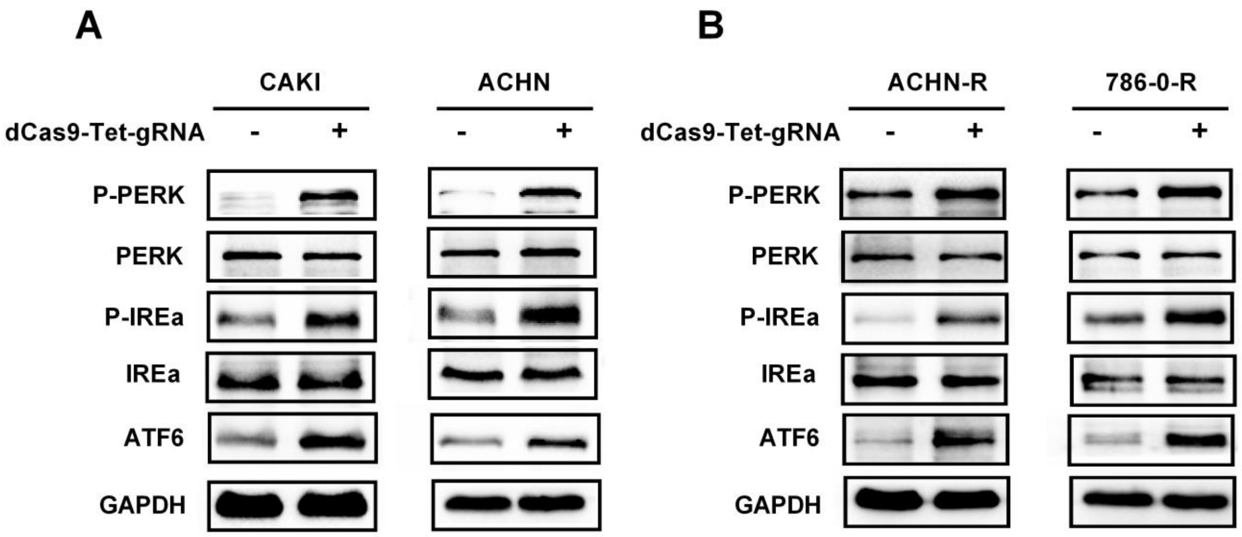

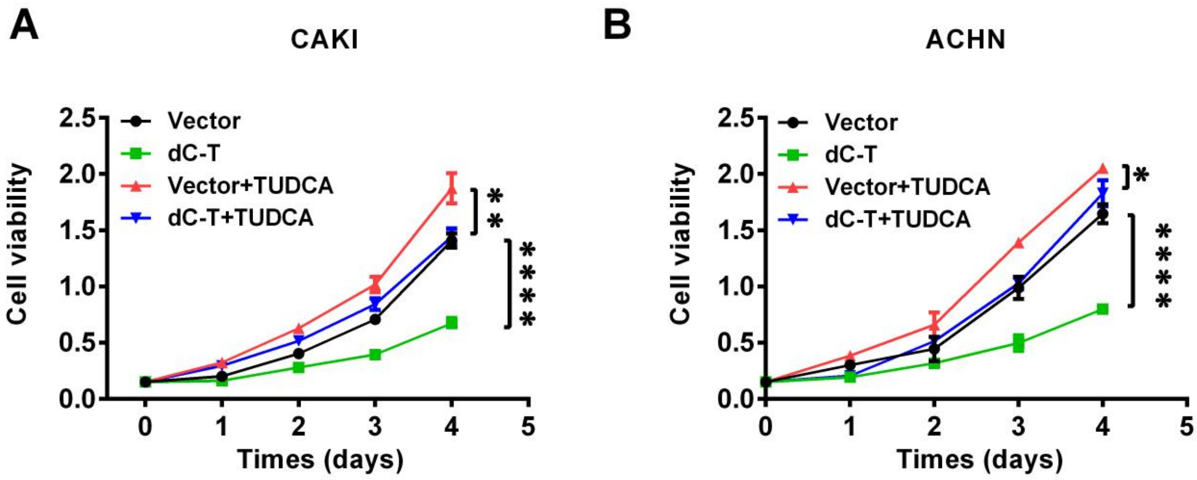
A
CAKI
Cell viability
Times (days)
Vector
dC-T
Vector+TUDCA
dC-T+TUDCA
**
****
B
ACHN
Cell viability
Times (days)
Vector
dC-T
Vector+TUDCA
dC-T+TUDCA
*
****

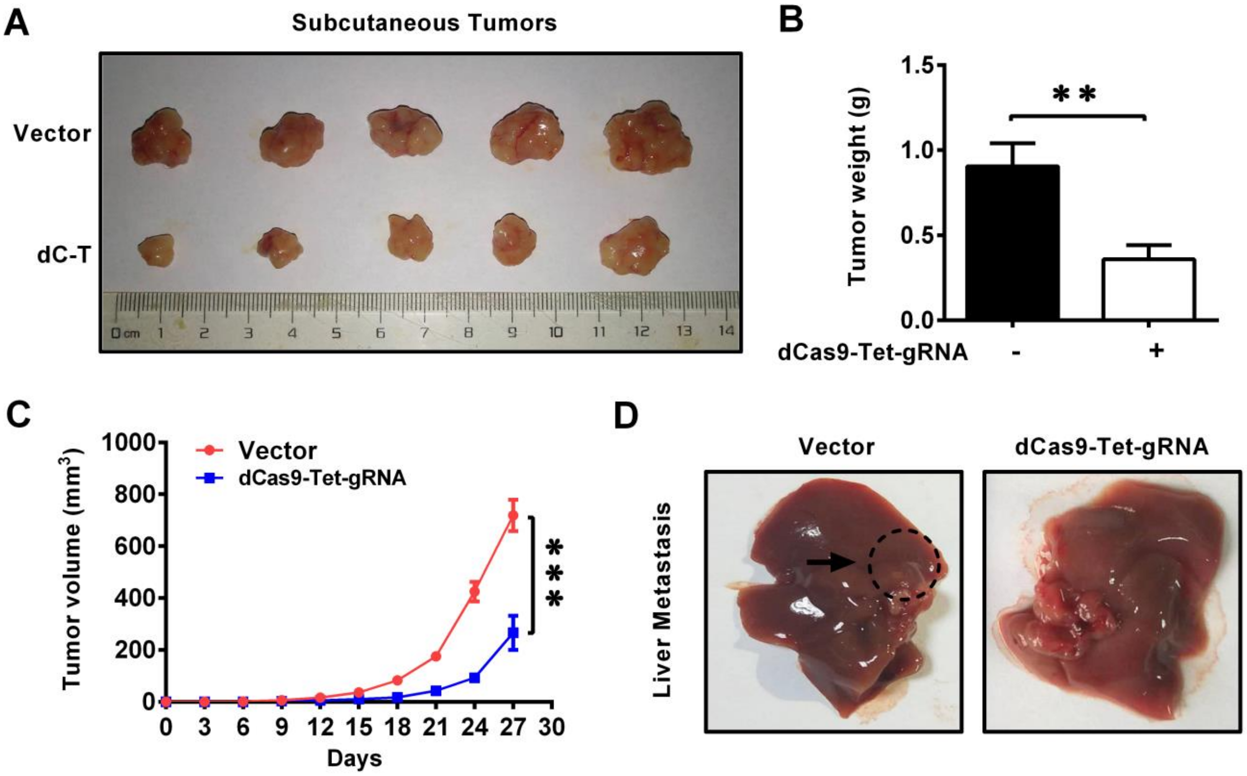
A
Subcutaneous Tumors
Vector
dC-T
B
Tumor weight (g)
1.5
1.0
0.5
0.0
**
dCas9-Tet-gRNA
-
+
C
Tumor volume (mm3)
1000
800
600
400
200
0
Vector
dCas9-Tet-gRNA
0 3 6 9 12 15 18 21 24 27 30
Days
***
D
Vector
dCas9-Tet-gRNA
Liver Metastasis
